# Caffeine protects against experimental acute pancreatitis by inhibition of inositol 1,4,5-trisphosphate receptor-mediated Ca^2+^ release

**DOI:** 10.1136/gutjnl-2015-309363

**Published:** 2015-12-07

**Authors:** Wei Huang, Matthew C Cane, Rajarshi Mukherjee, Peter Szatmary, Xiaoying Zhang, Victoria Elliott, Yulin Ouyang, Michael Chvanov, Diane Latawiec, Li Wen, David M Booth, Andrea C Haynes, Ole H Petersen, Alexei V Tepikin, David N Criddle, Robert Sutton

**Affiliations:** 1 NIHR Liverpool Pancreas Biomedical Research Unit, Royal Liverpool University Hospital, University of Liverpool, Liverpool, UK; 2 Department of Cellular and Molecular Physiology, Institute of Translational Medicine, University of Liverpool, Liverpool, UK; 3 Department of Integrated Traditional Chinese and Western Medicine, Sichuan Provincial Pancreatitis Centre, West China Hospital, Sichuan University, Chengdu, China; 4 Immuno-Inflammation Therapeutic Area Unit, GlaxoSmithKline, Stevenage, UK; 5 Cardiff School of Biosciences, Cardiff University, Cardiff, UK

**Keywords:** ACUTE PANCREATITIS, CALCIUM, DRUG DEVELOPMENT

## Abstract

**Objective:**

Caffeine reduces toxic Ca^2+^ signals in pancreatic acinar cells via inhibition of inositol 1,4,5-trisphosphate receptor (IP_3_R)-mediated signalling, but effects of other xanthines have not been evaluated, nor effects of xanthines on experimental acute pancreatitis (AP). We have determined effects of caffeine and its xanthine metabolites on pancreatic acinar IP_3_R-mediated Ca^2+^ signalling and experimental AP.

**Design:**

Isolated pancreatic acinar cells were exposed to secretagogues, uncaged IP_3_ or toxins that induce AP and effects of xanthines, non-xanthine phosphodiesterase (PDE) inhibitors and cyclic adenosine monophosphate and cyclic guanosine monophosphate (cAMP/cGMP) determined. The intracellular cytosolic calcium concentration ([Ca^2+^]_C_), mitochondrial depolarisation and necrosis were assessed by confocal microscopy. Effects of xanthines were evaluated in caerulein-induced AP (CER-AP), taurolithocholic acid 3-sulfate-induced AP (TLCS-AP) or palmitoleic acid plus ethanol-induced AP (fatty acid ethyl ester AP (FAEE-AP)). Serum xanthines were measured by liquid chromatography-mass spectrometry.

**Results:**

Caffeine, dimethylxanthines and non-xanthine PDE inhibitors blocked IP_3_-mediated Ca^2+^ oscillations, while monomethylxanthines had little effect. Caffeine and dimethylxanthines inhibited uncaged IP_3_-induced Ca^2+^ rises, toxin-induced Ca^2+^ release, mitochondrial depolarisation and necrotic cell death pathway activation; cAMP/cGMP did not inhibit toxin-induced Ca^2+^ rises. Caffeine significantly ameliorated CER-AP with most effect at 25 mg/kg (seven injections hourly); paraxanthine or theophylline did not. Caffeine at 25 mg/kg significantly ameliorated TLCS-AP and FAEE-AP. Mean total serum levels of dimethylxanthines and trimethylxanthines peaked at >2 mM with 25 mg/kg caffeine but at <100 µM with 25 mg/kg paraxanthine or theophylline.

**Conclusions:**

Caffeine and its dimethylxanthine metabolites reduced pathological IP_3_R-mediated pancreatic acinar Ca^2+^ signals but only caffeine ameliorated experimental AP. Caffeine is a suitable starting point for medicinal chemistry.

**Significance of this study**

**What is already known on this subject?**

- Acute pancreatitis is a major health problem without specific drug therapy.
- Coffee consumption reduces the incidence of acute alcoholic pancreatitis.
- Caffeine blocks physiological intracellular Ca^2+^ oscillations by inhibition of inositol 1,4,5-trisphosphate receptor-(IP_3_R)-mediated signalling.
- Sustained cytosolic Ca^2+^ overload from abnormal Ca^2+^ signalling is implicated as a critical trigger in the pathogenesis of acute pancreatitis.

**What are the new findings?**

- Caffeine and its dimethylxanthine metabolites inhibit IP_3_R-mediated, sustained cytosolic Ca^2+^ elevations, loss of mitochondrial membrane potential and necrotic cell death pathway activation in pancreatic acinar cells.
- Neither specific phosphodiesterase inhibitors nor cyclic adenosine monophosphate and cyclic guanosine monophosphate inhibit sustained Ca^2+^ elevations in pancreatic acinar cells.
- Serum levels of xanthines after 25 mg/kg caffeine administration are sufficient to inhibit IP_3_R-mediated Ca^2+^ overload in experimental acute pancreatitis.
- Caffeine but not theophylline or paraxanthine administered at 25 mg/kg significantly ameliorated pancreatic injury in experimental acute pancreatitis through IP_3_R-mediated signalling inhibition.

**How might it impact on clinical practice in the foreseeable future?**

- These findings support an approach of inhibition of Ca^2+^ overload and of its consequences as novel potential therapy for acute pancreatitis.
- Methylxanthine-based structures are suitable starting points for drug discovery and development to treat acute pancreatitis.

## Introduction

Acute pancreatitis (AP) has an incidence of 30 per 100 000 per annum in the UK, commonly caused by gallstones or alcohol excess.^1^ Most cases are mild, whereas a complicated clinical course occurs in one out of every five patients, resulting in significant morbidity, mortality and financial burden.^2^ Over the last two decades, our understanding of pathogenesis has advanced, but there is still no specific therapy despite many randomised trials.^2^ The development of treatments for AP is, therefore, a priority, one strategy for which is to follow leads from complementary laboratory and clinical studies, as here.

Intracellular Ca^2+^ signals control normal secretion from pancreatic acinar cells but can become a critical trigger in pathogenesis. Physiological concentrations of acetylcholine (ACh) and cholecystokinin (CCK) generate repetitive elevations in the cytosolic Ca^2+^ concentration ([Ca^2+^]_C_) within the cellular apical pole that elicit stimulus metabolism coupling to generate ATP from mitochondria and stimulus-secretion coupling to initiate exocytosis.^3^ Intermittently, global extension of short-lived signals throughout the cell is necessary for nuclear signalling contributing to transcription and translation.^3^ In contrast, toxins such as bile acids,^4^ oxidative^5^ and non-oxidative metabolites^6^ ^7^ of ethanol and CCK hyperstimulation^8^ ^9^ each elicit abnormal elevations of [Ca^2+^]_C_ that are global and sustained. These abnormal elevations induce premature activation of intracellular enzymes, mitochondrial dysfunction, impaired autophagy, vacuolisation and necrosis, all of which contribute to the pathogenesis of AP.^10^ Ca^2+^ chelation prevents zymogen activation and vacuolisation through attenuation of Ca^2+^ overload in acinar cells in vitro^11^ ^12^ and ameliorates the severity of AP in vivo.^13^ Blockage of the Ca^2+^ release-activated Ca^2+^ channel, also known as the store-operated Ca^2+^ entry (SOCE) channel, by Orai1 inhibitor GSK-7975A, reduces Ca^2+^ overload and necrosis in both mouse^14^ ^15^ and human^15^ pancreatic acinar cells and prevents AP in three different mouse models. Genetic deletion or pharmacological inhibition of another SOCE channel, transient receptor potential cation channel 3 (TRPC3), also reduces caerulein-induced SOCE and AP.^16^ ^17^

Excessive Ca^2+^ release from intracellular stores occurs predominantly via inositol 1,4,5-trisphosphate receptor (IP_3_R) Ca^2+^ channels.^18^ The pancreatic acinar cell expresses all three subtypes of the IP_3_R in the apical region, close to the luminal membrane,^19–21^ but IP_3_R types 2 and 3 are predominantly responsible for physiological Ca^2+^ signalling and enzyme secretion.^20^ Stimuli such as CCK,^22^ the bile acid taurolithocholic acid 3-sulfate (TLCS),^23^ ^24^ alcohol^25^ and fatty acid ethyl ester (FAEE)^6^ ^18^ cause intracellular Ca^2+^ release in pancreatic acinar cells primarily via IP_3_Rs, an effect inhibited by double knockout of IP_3_R types 2 and 3^18^ or by caffeine.^8^ ^18^

Caffeine (1,3,7-trimethyxanthine) belongs to the methylxanthine class of small, purine-based planar molecules and has several pharmacological actions,^26^ including pronounced actions on Ca^2+^ signalling.^27^ Caffeine inhibits Ca^2+^ release from IP_3_Rs by inhibition of phospholipase C-mediated production of IP_3_^28^ or by antagonising IP_3_Rs^29^ through direct binding and reduction of the open-state probability of IP_3_Rs.^30^ ^31^ Contrarily, caffeine activates Ca^2+^ release from ryanodine receptors (RyRs) by increasing the sensitivity of RyRs to Ca^2+^ itself as observed in multiple cells,^32^ although in pancreatic acinar cells effects on IP_3_Rs predominate.^28^ ^29^

The effects of caffeine on IP_3_-mediated Ca^2+^ signalling may be protective in AP since the incidence of AP is inversely proportional to the amount of coffee consumed.^33^ Caffeine also inhibits cyclic adenosine monophosphate (cAMP) and cyclic guanosine monophosphate (cGMP) phosphodiesterase (PDE), which degrades cAMP and cGMP to non-cyclic forms;^34^ inhibition of PDE reduces tumour necrosis factor and leukotriene synthesis, inhibiting innate immunity.^35^ Caffeine is a non-selective inhibitor of adenosine receptors, removing an endogenous brake on neural activity.^26^ This stimulant effect of caffeine is the most familiar, but taken to excess may result in caffeine intoxication with major central nervous system hyperstimulation.^26^ Degradation of caffeine in the liver forms the dimethylxanthines theophylline (1,3-dimethylxanthine), paraxanthine (1,7-dimethylxanthine) and theobromine (3,7-dimethylxanthine), used variously as drugs with similar actions to those of caffeine, although their actions on IP_3_R-mediated signalling have not been clarified. As data suggest caffeine and/or related methylxanthines may be protective in AP, we sought to determine their actions on toxin-induced, IP_3_R-mediated [Ca^2+^]_C_ changes and cell death in vitro, and in three models of AP in vivo.

## Materials and methods

### Animals

Adult male CD1 mice (8–12 weeks old) were housed at 23±2°C under a 12 h light/dark cycle with ad libitum access to standard laboratory chow and water. For in vivo experiments, animals were deprived of food but were allowed access to water from 12 h before the start of the experiments.

### Measurements of Ca^2+^ responses, mitochondrial membrane potential (ΔΨ_M_) and IP_3_ uncaging

Fresh pancreatic acinar cells were isolated as described.^7^ Fluo 4-AM (3 μM), ci-IP_3_/PM (2 µM) and/or tetramethyl rhodamine methyl ester (TMRM, 37.5 nM) were loaded for 30 min at room temperature. Confocal images were acquired on a Zeiss LSM510 system (Carl Zeiss Jena GmbH, Germany) with a 63× C-Apochromat water immersion objective (NA 1.2). ΔΨ_M_ was recorded in the perigranular mitochondrial cell region. IP_3_ was uncaged by UV excitation of whole cells (364 nm, 1% power) every three seconds where indicated. All fluorescence measurements were expressed as changes from basal fluorescence (F/F_0_ ratio), where F_0_ represents initial fluorescence at the start of each experiment.

### In vitro necrosis assays

For CCK-induced cell death, a time-course propidium iodide (50 µM) necrosis assay was run at 37°C using a POLARstar Omega Plate Reader (BMG Labtech, Germany). Isolated murine pancreatic acinar cells (75 µL) were added to a caffeine solution (75 µL) at selected concentrations or the same volume of physiological saline (for controls) prior to CCK (50 nM) addition.

In TLCS-induced cell injury, an end-point propidium iodide (100 µg/mL) necrosis assay was employed. Cells were incubated with respective test solutions and agitated by rotary inversion for 30 min at 37°C, centrifuged (at 260 *g* for 2 min), resuspended and transferred to a microplate. Data were calculated as background-subtracted (cell-free blanks) percentage of total death (in 0.02% TritonX). Data were normalised to minimum and maximum fluorescence using the formula (F-F_max_)/(F_max_ − F_min_)+1. All experiments were in triplicate.

### Determination of serum dimethylxanthine and trimethylxanthine levels by liquid chromatography-mass spectrometry

Serum was analysed on a QTRAP5500 hybrid triple-quadrupole/linear ion trap instrument with TurboIon V Ion source (Applied Biosystems, UK), with inline LC (Ultimate 3000 (Thermoscientific/Dionex, UK)) and Gemini C18, 3 µm, 2.1×100 mm column (Phenomenex, UK). Eluent A comprised H_2_O/0.1%, formic acid (FA)/1% and *tetrahydrofuran* v/v, Eluent B 100% acetonitrile/0.1% FA v/v. The QTRAP5500 was operated in positive electrospray ionisation (ESI) mode and two MRM transitions were monitored for caffeine (195.3/138.0 and 195.3/110.0), theobromine (181.1/124.0 and 181.1/96.0), paraxanthine (181.2/124.0 and 181.2/142.0), theophylline (181.7/96.0 and 181.7/124.0) and internal standard (paracetamol—152.064/110.0 and 152.064/65.0) with a 100 ms dwell time. Also, 1 µL of 100 µM internal standard was added to 50 µL of each mouse serum sample and subjected to acetone precipitation (8:1 v/v) at −20°C for 1 h. Samples were centrifuged at 14 000*g* for 10 min at 4°C, then supernatant vacuum centrifuged to a volume of 50 µL. A 10 µL aliquot was injected into the liquid chromatography-mass spectrometry system. All xanthine serum concentrations were determined using a calibration curve of 1–100 µM for each analyte, spiked in mouse serum.

### Experimental AP

Hyperstimulation AP was induced by either 7 or 12 intraperitoneal injections of 50 µg/kg caerulein hourly (CER-AP), with saline controls. Bile acid AP was induced by retrograde infusion of 50 µL taurolithocholate acid sulfate (3 mM, TLCS-AP) into the pancreatic duct as described, with saline injection (sham) controls.^10^ ^36^ FAEE-AP was induced by simultaneous intraperitoneal injection of ethanol (1.35 g/kg) and palmitoleic acid (POA, 150 mg/kg), twice at 1 h apart.^7^ Control mice received only ethanol (1.35 g/kg) injections. In all models, analgesia with 0.1 mg/kg buprenorphine hydrochloride (Temgesic, Reckitt and Coleman, Hull, England) was administered. Mice were humanely killed at designated time points for determination of severity (see online supplementary materials and methods).

### Caffeine administration in vivo

Details of caffeine dose optimisation and administration of other methylxanthines are described in supplementary materials and methods. In CER-AP, mice received seven intraperitoneal injections of 1, 5, 10 or 25 mg/kg of caffeine (called regimen subsequently) hourly, beginning 2 h after the first caerulein injection, and were humanely killed at 12 h for determination of severity. The effect of caffeine was also assessed in both 7-injection and 12-injection CER-AP models at 24 h. In TLCS-AP, caffeine (25 mg/kg regimen) was begun 1 h after TLCS infusion and severity determined after humane killing at 24 h. In FAEE-AP, two intraperitoneal injections of caffeine (25 mg/kg, 1 h apart) were administered from an hour after the second POA/ethanol injection.

### Statistical analysis

Results are presented as means±SEM from three or more independent experiments. In all figures, vertical bars denote mean±SE values. Statistical analysis was performed using Student's t test or analysis of variance in Origin 8.5 (OriginLab, Northampton, Massachusetts, USA) and a value of p<0.05 considered significant.

### Chemicals

Fluo 4-AM, TMRM and Hoechst 33342 were from Thermo Fisher Scientific (Waltham, Massachusetts, USA); ci-IP_3_/PM from SiChem GmbH (Bremen, Germany). Unless otherwise stated, all other chemicals were from Sigma (Gillingham, UK) of the highest grade available.

## Results

### Inhibition of ACh-induced [Ca^2+^]_C_ oscillations by caffeine and its dimethylxanthine metabolites

**Figure 1:**
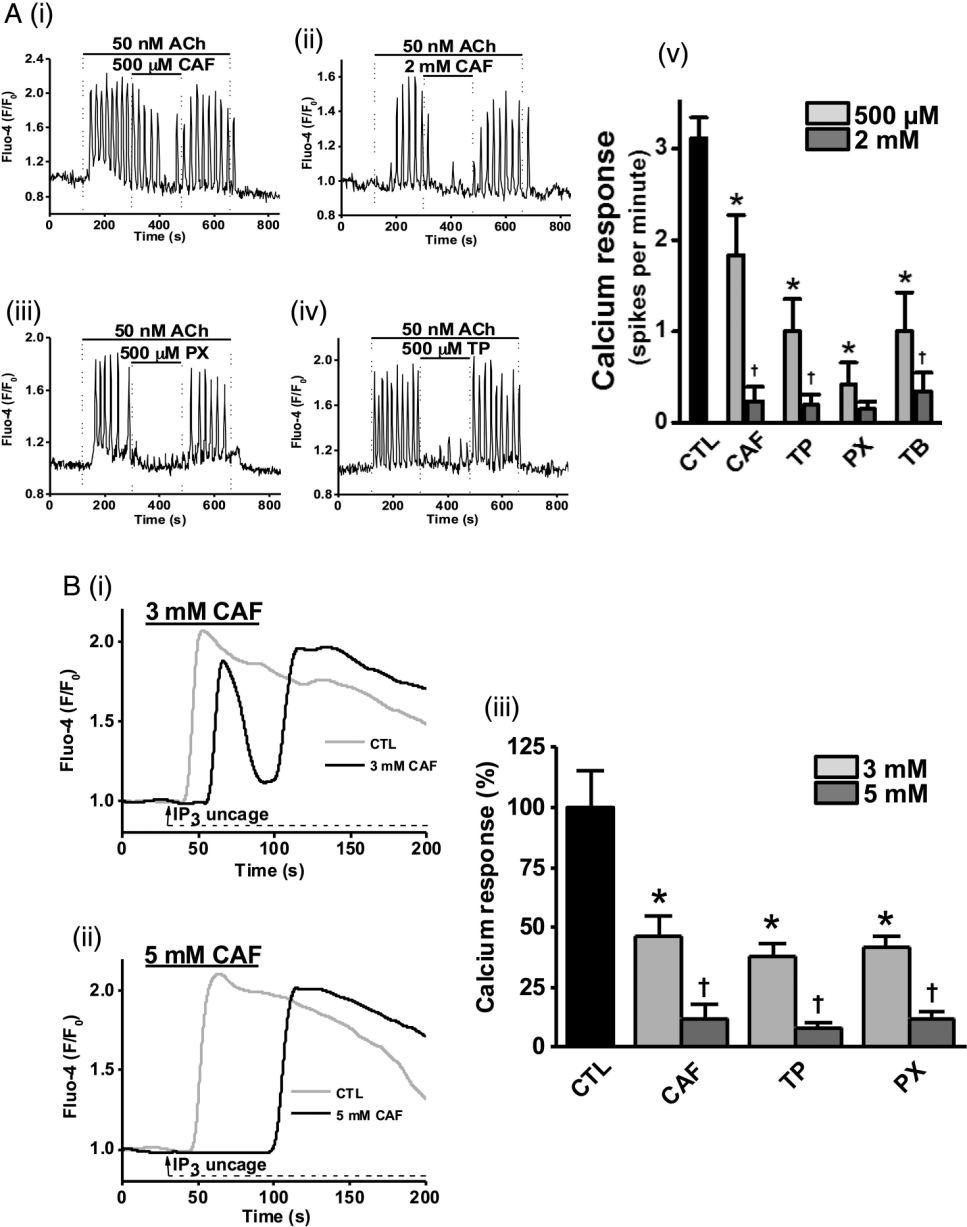
Dimethylxanthine and trimethylxanthines inhibit acetylcholine (ACh)-induced and inositol 1,4,5-trisphosphate receptor (IP_3_)-induced Ca^2+^ signals in isolated pancreatic acinar cells. (A) Representative traces of ACh (50 nM) induced Ca^2+^ oscillations that were significantly inhibited by caffeine (CAF), theophylline (TP) and paraxanthine (PX): (i) partial inhibition by CAF at 500 µM, (ii) almost complete inhibition by CAF at 2 mM, or (iii) TP at 500 µM or (iv) PX at 500 µM. (v) Summary histograms of the inhibitory effects of CAF, TP, PX and theobromine (TB) on ACh-induced Ca^2+^ oscillations at both 500 µM and 2 mM. (B) Representative traces of Ca^2+^ elevations (grey) generated by uncaging of the membrane permeable IP_3_ analogue, ci-IP_3_/PM (2 µM) that were significantly inhibited by CAF (black): (i) partial inhibition at 3 mM and (ii) complete inhibition at 5 mM. (iii) Summary histograms of inhibitory effects of CAF, TP and PX on IP_3_-induced Ca^2+^ elevations at 3 and 5 mM. *p<0.05 vs control group; †p<0.05 vs lower concentration. Traces are averages of >20 cells from at least three repeat experiments. Data normalised from basal fluorescence levels (F/F_0_) and are expressed as means±SE in histograms.

ACh (50 nM) caused [Ca^2+^]_C_ oscillations in pancreatic acinar cells that were concentration-dependently inhibited by caffeine at 500 µM to 2 mM (figure 1Ai, ii); 200 µM caffeine resulted in no significant reduction (data not shown). ACh-induced [Ca^2+^]_C_ oscillations were also inhibited by 500 µM theophylline (figure 1Aiii) and 500 µM paraxanthine (figure 1Aiv); all dimethylxanthines inhibited ACh-induced [Ca^2+^]_C_ signals in a concentration-dependent manner (figure 1Av). Theophylline, paraxanthine and theobromine induced significantly more inhibition than caffeine at 500 µM, with paraxanthine showing the highest potency. In contrast, 1-methylxanthine and xanthine showed minimal inhibition (see online supplementary figure S1A, B).

### Inhibition of IP_3_-mediated [Ca^2+^]_C_ signals by caffeine and its dimethylxanthine metabolites

To investigate whether methylxanthines might directly inhibit IP_3_R-mediated Ca^2+^ elevations, a membrane-permeable caged IP_3_ analogue, ci-IP_3_/PM, was loaded into pancreatic acinar cells. Repetitive uncaging of ci-IP_3_/PM caused sustained increases of [Ca^2+^]_C_ that were inhibited in a concentration-dependent manner by caffeine (3 and 5 mM) (figure 1Bi, ii). Theophylline and paraxanthine showed similar effects (figure 1Biii). These results suggest that methylxanthines inhibit IP_3_R-mediated [Ca^2+^]_C_ signals by an action on the IP_3_R.

### Caffeine-induced inhibition of CCK-induced [Ca^2+^]_C_ signals, ΔΨ_M_ loss and cell death

**Figure 2:**
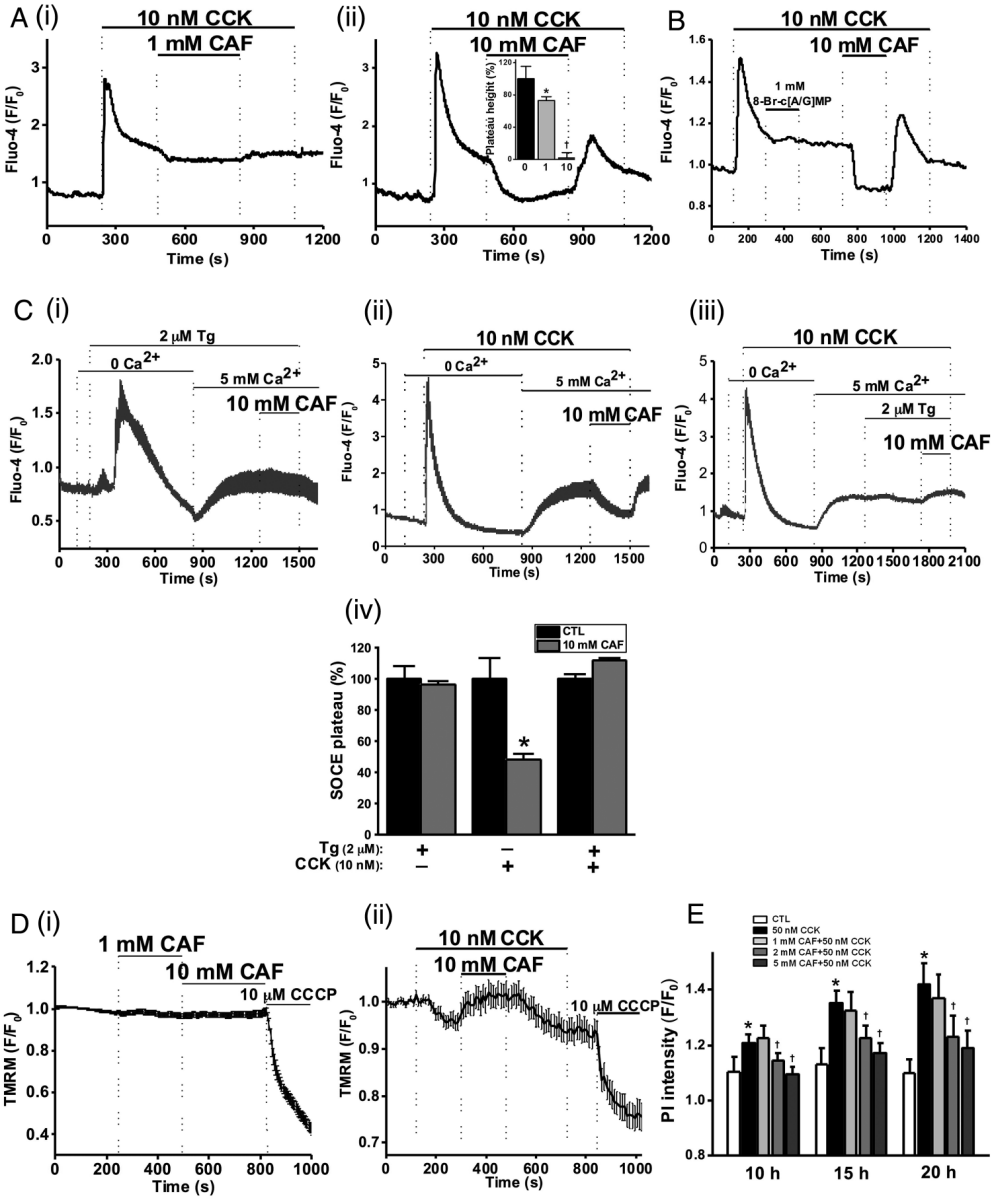
Caffeine (CAF) inhibits cholecystokinin (CCK)-induced sustained Ca^2+^ signals, mitochondrial membrane potential (ΔΨ_M_) loss and cell death. (A) Representative traces showing the CCK-induced (10 nM) Ca^2+^ plateau that was significantly inhibited by CAF: (i) partial inhibition at 1 mM and (ii) almost complete inhibition at 10 mM, with mean plateau height as % above baseline (inset) showing CAF has a dose-dependent inhibitory effect on the plateau height (*p<0.05 vs control group; †p<0.05 vs lower concentration). (B) Representative trace showing the lack of inhibitory effect of non-hydrolysable analogues of cyclic adenosine monophosphate (cAMP) and cyclic guanosine monophosphate (cGMP), 8-bromo-cAMP/cGMP (1 mM) on the CCK-induced Ca^2+^ plateau, subsequently abolished by CAF (10 mM). (C) Representative traces and summary histogram showing that CAF (10 mM) (i) did not inhibit the store-operated Ca^2+^ entry plateau (SOCE) induced by thapsigargin (TG, 2 µM) but (ii) did inhibit SOCE induced by CCK (10 nM); (iii) CAF did not inhibit SOCE in the presence of TG. (iv) Summary histogram of the effect of CAF on the SOCE plateau in the presence of TG, CCK, or both (*p<0.05 vs control group). (D) Loss of mitochondrial ΔΨ_M_ (tetramethyl rhodamine methyl, TMRM) induced by CCK (10 nM) was reversed by application of CAF (10 mM), after removal of which the ΔΨ_M_ dropped once more and addition of the protonophore (CCCP, 10 µM) collapsed this to a minimal level: (i) CAF itself had no significant effect on ΔΨ_M_; (ii) effect on CAF on CCK-induced ΔΨ_M_ loss. (E) CAF significantly inhibited necrotic cell death pathway activation (PI uptake) induced by CCK (50 nM) in a dose-dependent manner at 2 and 5 mM (*p<0.05 vs control group; †p<0.05 vs CCK only). Traces are averages of >19 cells from at least three repeat experiments. Data normalised from basal fluorescence levels (F/F_0_) and are expressed as means±SE in histograms.

The effects of caffeine on CCK-induced toxic, sustained [Ca^2+^]_C_ signals were investigated. An elevated Ca^2+^ plateau followed hyperstimulation with 10 nM CCK (figure 2A), which was reduced by 27% by 1 mM caffeine (figure 2Ai), and blocked by 10 mM (figure 2Aii), effects reversible upon washout. Similar effects were observed when 10 mM caffeine was applied prior to 10 nM CCK stimulation (see online supplementary figure S2A).

Methylxanthines are PDE inhibitors and simultaneous increases in cAMP and cGMP may synergistically inhibit [Ca^2+^]_C_ oscillations induced by ACh.^37^ The potential contribution of PDE inhibition to the effects of caffeine on CCK-induced sustained Ca^2+^ signals was investigated using non-hydrolysable analogues of cAMP and cGMP. Addition of 8-bromo-cAMP/GMP (1 mM) did not affect the CCK-induced [Ca^2+^]_C_ plateau, whereas 10 mM caffeine caused complete inhibition (figure 2B), suggesting a mechanism independent of intracellular cyclic nucleotide levels, although both xanthine and non-xanthine PDE inhibitors were found to inhibit ACh-induced [Ca^2+^]_C_ oscillations (see online supplementary figure S3A–D).

To test potential effects of caffeine on SOCE, internal Ca^2+^ stores were depleted under Ca^2+^-free conditions using either 10 nM CCK or 2 µM thapsigargin, an inhibitor of the sarco-endoplasmic reticulum calcium transport ATPase (SERCA) and SOCE triggered by reapplication of extracellular Ca^2+^ (5 mM). Following depletion of internal stores with thapsigargin, caffeine was unable to revert the SOCE-induced Ca^2+^ plateau (figure 2Ci). When 10 nM CCK was used to deplete internal stores, the sustained SOCE plateau was significantly inhibited by 10 mM caffeine in a reversible manner (figure 2Cii). Following application of both CCK and thapsigargin, caffeine did not reduce the associated SOCE (figure 2Ciii). These data, summarised in figure 2Civ, are consistent with an inhibitory action of caffeine on IP_3_R-mediated signalling, not SOCE per se.

Since sustained [Ca^2+^]_C_ elevations are known to induce mitochondrial dysfunction leading to pancreatic acinar cell necrosis,^6^ ^7^ ^10^ the effects of caffeine on ΔΨ_M_ were also evaluated. Caffeine (both 1 and 10 mM) did not significantly affect ΔΨ_M_ on its own (figure 2Di), but it (10 mM) inhibited the loss of ΔΨ_M_ induced by CCK, reversible on removal of the xanthine (figure 2Dii). In a time-course necrotic cell death pathway activation assay, caffeine (2 and 5 mM) reduced 50 nM CCK-induced cell death in a concentration-dependent and time-dependent manner (figure 2E).

### Inhibition of TLCS-induced [Ca^2+^]_C_ signals and cell death by caffeine and its dimethylxanthine metabolites

**Figure 3:**
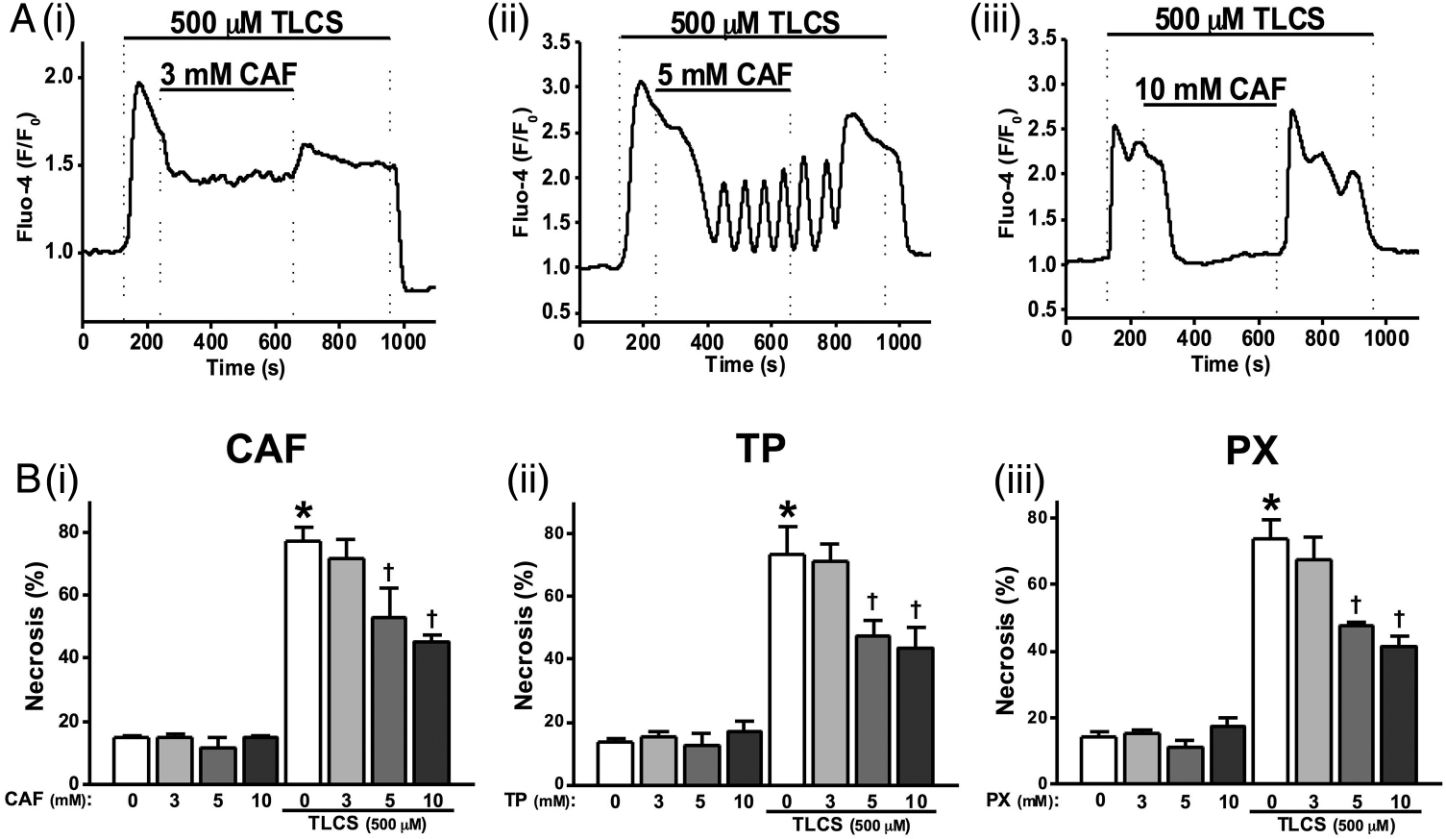
Effects of methylxanthines on taurolithocholic acid 3-sulfate (TLCS)-induced Ca^2+^ signals and cell death. (A) Representative traces showing that the TLCS-induced (500 µM) Ca^2+^ plateau was significantly inhibited by caffeine (CAF): (i) partial inhibition at 3 mM, (ii) the sustained Ca^2+^ plateau was converted to oscillations at 5 mM and (iii) complete inhibition at 10 mM. (B) (i) CAF significantly inhibited necrotic cell death pathway activation (PI uptake) induced by TLCS (500 µM) in a dose-dependent manner at 5 and 10 mM. Similar effects were also seen for (ii) theophylline (TP) and (iii) paraxanthine (PX). CAF, TP and PX did not affect basal PI uptake compared with normal controls (*p<0.05 vs control group; †p<0.05 vs TLCS only). Traces are averages of >20 cells from at least three repeat experiments. Data normalised from basal fluorescence levels (F/F_0_) for Ca^2+^ signals and from maximal fluorescence levels (F/F_max_) for PI uptake, respectively. Data are expressed as means±SE in histograms.

To investigate effects of caffeine on bile acid induced [Ca^2+^]_C_ signals, 500 µM TLCS was applied to induce sustained [Ca^2+^]_C_ elevations in pancreatic acinar cells. Caffeine concentration-dependently blocked these TLCS-induced [Ca^2+^]_C_ elevations. Thus, 3 mM caffeine partially reduced the plateau (figure 3Ai), 5 mM caffeine further reduced the sustained elevation with oscillatory [Ca^2+^]_C_ rises sometimes superimposed (figure 3Aii), while 10 mM completely blocked the sustained elevations (figure 3Aiii). Pretreatment of cells with 10 mM caffeine converted 500 µM TLCS-induced [Ca^2+^]_C_ plateaus into oscillations (see online supplementary figure S2B).

The effects of methylxanthines on TLCS-induced necrosis were investigated using an end-point assay. Caffeine, theophylline and paraxanthine concentration-dependently inhibited TLCS-induced toxicity (figure 3Bi–iii). Caffeine induced a slight but significant reduction of TLCS-induced necrosis at 5 mM and approximately halved this at 10 mM (figure 3Bi). Similar patterns were observed for theophylline and paraxanthine over the range of concentrations tested (figure 3Bii, iii).

### Serum dimethylxanthine and trimethylxanthine levels in CER-AP

**Figure 4:**
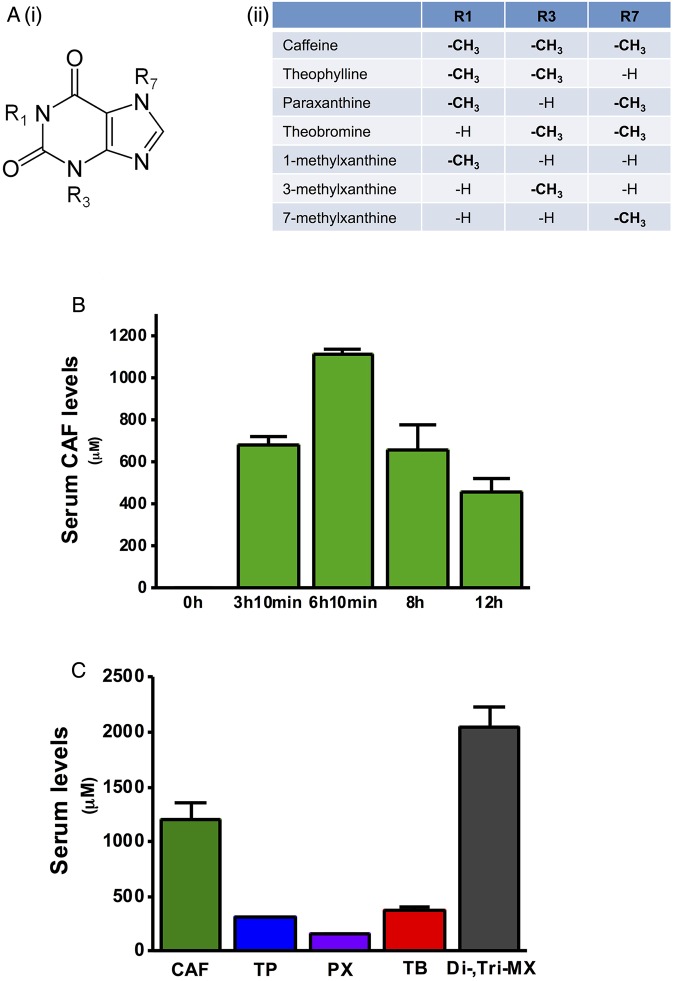
Methylxanthine (MX) structure and determination of serum di-MX and tri-MX levels in caerulein acute pancreatitis (CER-AP). (A) (i) Positions 1, 3 and 7 methylation of the xanthine structure are shown. (ii) Dependent on methylation state, caffeine (CAF) and its MX metabolites are classed as mono-MX, di-MX and tri-MX that are listed in the table. (B) In CER-AP, caffeine at 25 mg/kg (seven injections hourly) was given simultaneously with each CER (50 µg/kg) injection. Mice were sacrificed at different time points to measure serum caffeine (CAF, tri-MX) levels by LC/MS. (C) Respective serum di-MX levels and total di-MX and tri-MX levels showing peak caffeine concentration at 10 min after last caffeine/CER injection: CAF had the highest serum concentration, followed by theobromine (TB), theophylline (TP) and paraxanthine (PX). The cumulative concentration of di-MX and tri-MX was >2 mM. Values are means±SE from six mice.

The major metabolites of caffeine that appear in the blood stream of both humans and rodents are theophylline, paraxanthine, theobromine and monomethylxanthines (figure 4A). The serum levels of these were measured following in vivo caffeine administration to mice (25 mg/kg regimen) during CER-AP. The serum levels of caffeine were up to 700 μM at 10 min after four caffeine injections (figure 4B). It peaked at 10 min after seven injections of caffeine at >1 mM and gradually reduced to >600 and >400 μM at 2 and 6 h after last caffeine injection, respectively (figure 4B). Caffeine was the most abundant xanthine detected (∼1200 µM 10 min after seven injections), followed by theobromine (∼400 µM), theophylline (∼300 µM) and paraxanthine (∼150 µM) (figure 4C). The total level of dimethylxanthine and trimethylxanthine rose to >2 mM, a concentration capable of exerting marked inhibition of CCK-induced Ca^2+^ signals and cell death.

### Effects of dimethylxanthine and trimethylxanthine on the severity of CER-AP

**Figure 5:**
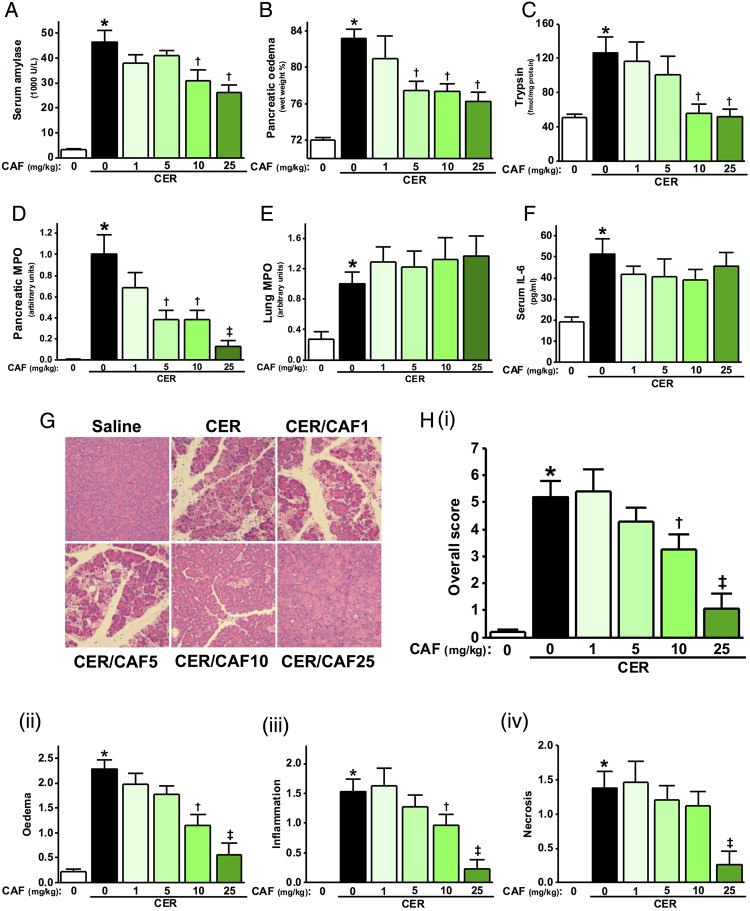
Dose-dependent protective effects of caffeine on the severity of caerulein acute pancreatitis (CER-AP) at 12 h. Mice received either intraperitoneal injections of 50 µg/kg CER (seven injections hourly) or equal amount of saline injections. Caffeine (CAF) at 1, 5, 10 or 25 mg/kg regimen (seven injections hourly) was begun 2 h after the first injection of CER. Mice were sacrificed at 12 h after disease induction and assessed for (A) serum amylase, (B) pancreatic oedema, (C) pancreatic trypsin activity, (D) pancreatic myeloperoxidase (MPO) activity (normalised to CER group), (E) lung MPO activity (normalised to CER group) and (F) serum interleukin (IL-6). (G) Representative images of pancreatic histopathology for all groups (H&E, ×200). (H) (i) Overall histopathological score and components: (ii) oedema, (iii) inflammation and (iv) necrosis. *p<0.05 vs control group; †p<0.05 vs CER group. Values are means±SE of 6–8 animals per group.

Since caffeine and its dimethylxanthine metabolites were able to protect against Ca^2+^-induced toxicity in vitro, an evaluation of caffeine was carried out in vivo on CER-AP. In the CER-AP with seven caerulein injections, at 12 h after the first caerulein injection there were significant elevations of serum amylase, pancreatic oedema (pancreatic wet to dry ratio), trypsin and myeloperoxidase (MPO) activity (a marker of neutrophil infiltration), with increases of lung MPO activity, alveolar membrane thickening and serum interleukin (IL)-6 (figure 5A–F and online supplementary figure S4A, B). To evaluate possible further distant organ injury, we assessed renal pathology in CER-AP, but no significant effects were seen on serum creatinine and renal histology, which appeared normal (see online supplementary figure S4C, D). Typical histopathological features of AP (oedema, vacuolisation, neutrophil infiltration and necrosis) were confirmed and mirrored by histopathology scores (figure 5G, H).

In agreement with in vitro findings, there was dose-dependency for caffeine in ameliorating the severity of CER-AP (figure 5A–F). Using 1 mg/kg caffeine regimen, there was no significant effect; with 5 mg/kg caffeine, there was significant reduction of pancreatic oedema and MPO activity, although other parameters remained unchanged. With 10 and 25 mg/kg caffeine regimens, there was marked suppression of serum amylase, pancreatic oedema, trypsin and MPO activity, whereas elevated lung MPO activity, alveolar membrane thickening and elevated serum IL-6 levels remained unsuppressed (figure 5A–F and online supplementary figure 4B). Caffeine had no significant effect on serum creatinine and renal histology (see online supplementary figure S4C, D). Caffeine at both 10 and 25 mg/kg markedly reduced the overall histopathology score (figure 5Hi). The protective effect at 25 mg/kg was the most marked (figure 5G), confirmed by the histopathological scores (figure 5Hii–iv). In other experimental AP models, the 25 mg/kg regimen was used, reduced to two injections for FAEE-AP.

To determine whether caffeine reduced pancreatic injury through direct vascular actions that increased blood flow,^38^ we determined pancreatic blood flow using fluorescent microspheres in untreated animals (see online supplementary materials and methods), in CER-AP and in CER-AP following 25 mg/kg caffeine regimen. While CER-AP markedly reduced pancreatic blood flow, caffeine did not have a significant effect on this flow, although there was a trend towards a modest improvement (see online supplementary figure S4E).

In contrast of the dramatic effects of caffeine on caerulein-induced pancreatic injury, theophylline and paraxanthine did not exert significant protective effects in CER-AP with both 10 and 25 mg/kg regimens (see online supplementary figure S5A–E). To further explore these unexpected findings, the serum levels of theophylline and paraxanthine were measured from both dose regimens. Serum levels of theophylline and paraxanthine 10 min after the last xanthine injection were each <100 µM in the 25 mg/kg regimen and <50 µM in the 10 mg/kg regimen (see online supplementary figure S6). These dimethylxanthine concentrations were previously shown not to alter IP_3_R-mediated [Ca^2+^]_C_ signals in vitro, consistent with an effect of caffeine on this signalling pathway.

**Figure 6:**
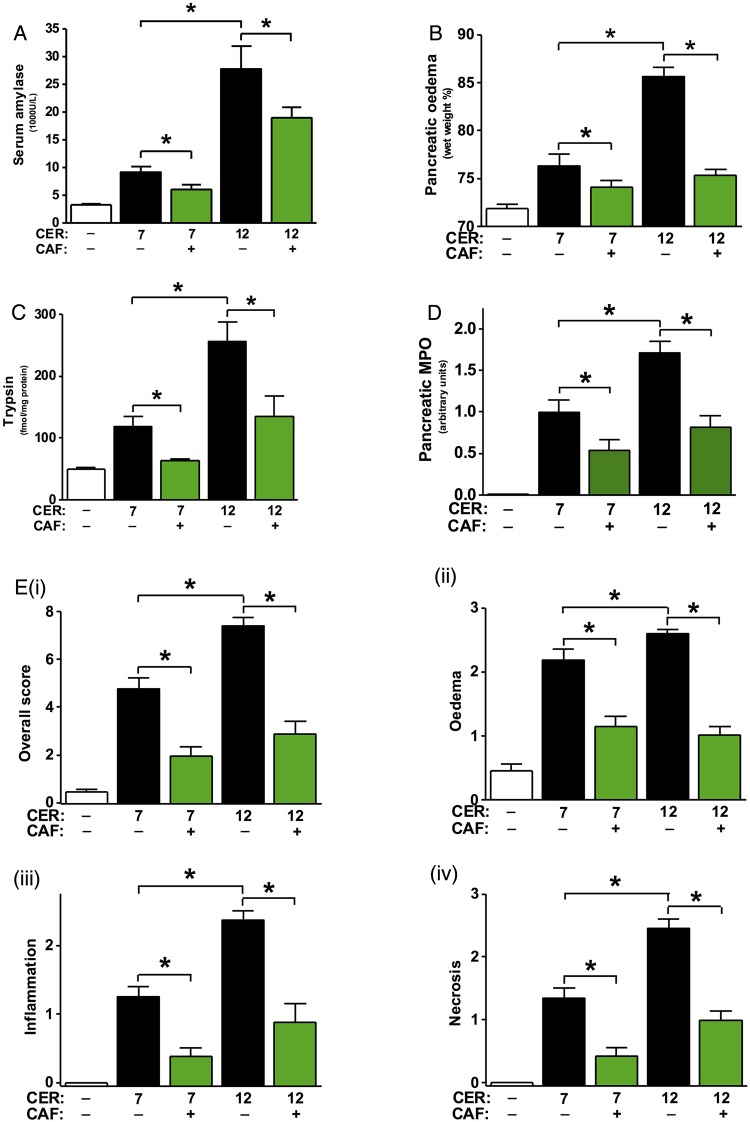
Caffeine (CAF) protects against pancreatic injury in two caerulein acute pancreatitis (CER-AP) models at 24 h. Mice received either intraperitoneal injections of 50 µg/kg CER (both 7 and 12 injections hourly) or equal amounts of saline injections. Caffeine (CAF) at the 25 mg/kg regimen (7 injections hourly) was begun 2 h after the first injection of CER. Mice were sacrificed at 24 h after disease induction and were assessed for (A) serum amylase, (B) pancreatic oedema, (C) pancreatic trypsin activity and (D) pancreatic myeloperoxidase (MPO) activity (normalised to CER group). (E) (i) Overall histopathological score and components: (ii) oedema, (iii) inflammation and (iv) necrosis. *Indicates p<0.05. Values are means±SE of 6–8 animals per group.

Since caffeine treatment was markedly protective in CER-AP at 12 h after induction by seven caerulein injections, its effects on more severe disease at a later time point were compared (figure 6). CER-AP induced by 12 hourly caerulein injections converted mild necrotising AP into a severe necrotising form characterised by extensive pancreatic oedema, neutrophil infiltration and necrosis at 24 h after induction (figure 6Ei–iv). Caffeine (25 mg/kg regimen) markedly reduced all parameters of pancreatic injury in both models.

### Protective effects of caffeine on TLCS-AP and FAEE-AP

**Figure 7:**
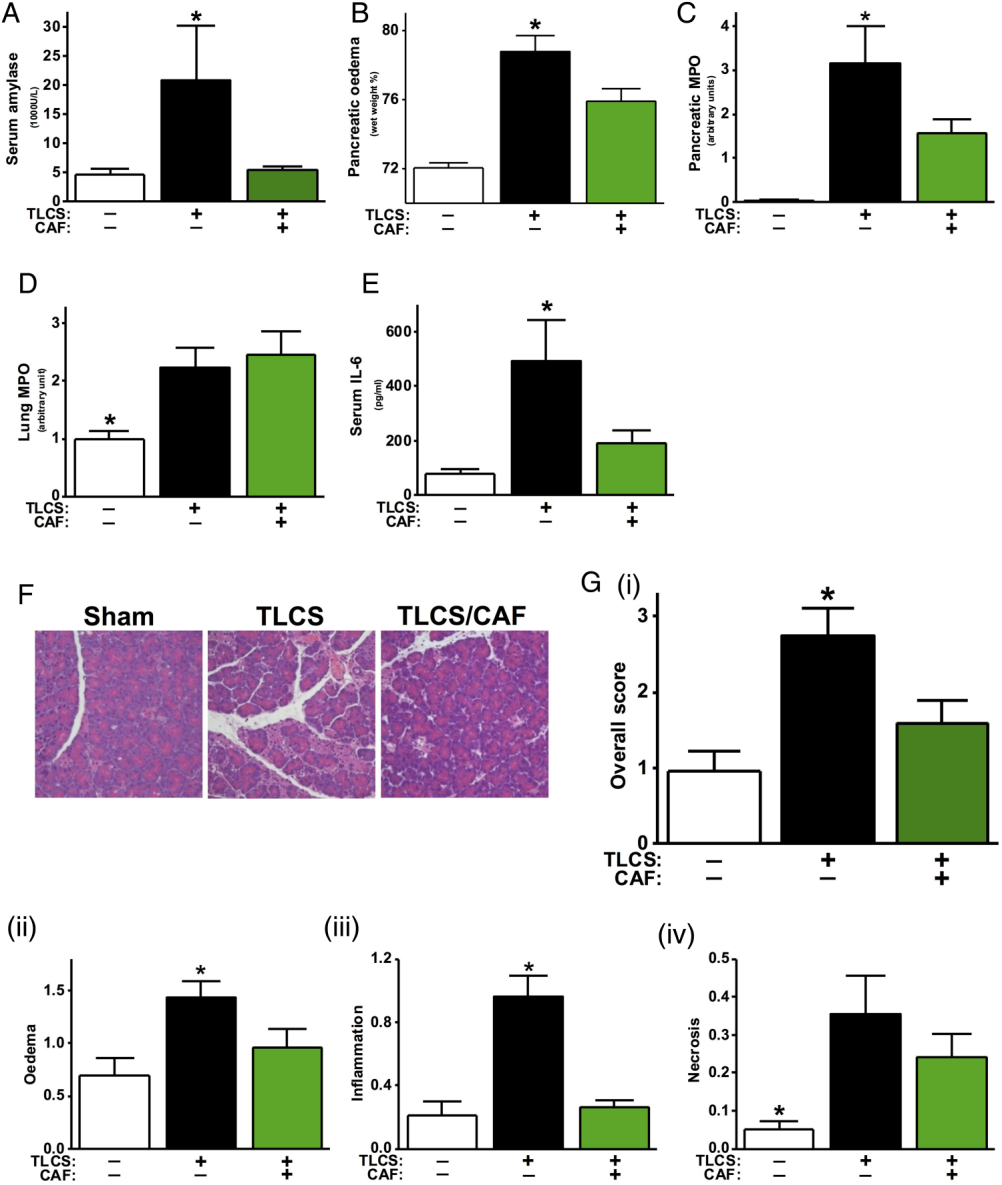
Protective effects of caffeine (CAF) on taurolithocholic acid 3-sulfate (TLCS)-acute pancreatitis (AP). Mice received either retrograde infusion of 50 µL of 3 mM TLCS into the pancreatic duct or underwent sham surgery. CAF at 25 mg/kg (seven injections hourly) was begun 1 h after TLCS infusion. Mice were sacrificed at 24 h after disease induction and were assessed for (A) serum amylase level, (B) pancreatic oedema, (C) pancreatic myeloperoxidase (MPO) activity (normalised to sham group), (D) lung MPO activity (normalised to sham group) and (E) serum interleukin (IL-6). (F) Representative pancreatic histopathology for all groups (H&E, ×200). (G) (i) Overall histopathological score and components: (ii) oedema, (iii) inflammation and (iv) necrosis. *p<0.05 vs other two groups. Values are means±SE of 6–11 animals per group.

TLCS-AP caused dramatic increases of pancreatic and systemic injury markers compared with the sham group at 24 h (figure 7A–E), with marked histopathological changes (figure 7F). Since pancreatic trypsin activity peaks very early after induction of AP in the bile acid-induced model, this parameter was not included for severity assessment.^36^ Caffeine significantly reduced serum amylase (figure 7A), pancreatic oedema (figure 7B), pancreatic MPO activity (figure 7C) and serum IL-6 (figure 7E), but did not affect lung MPO activity (figure 7D). Caffeine significantly reduced the overall histopathological score (figure 7Gi), as well as the specific oedema (figure 7Gii) and inflammation scores (figure 7Giii), with a trend to curtail the necrosis score (figure 7Giv).

**Figure 8:**
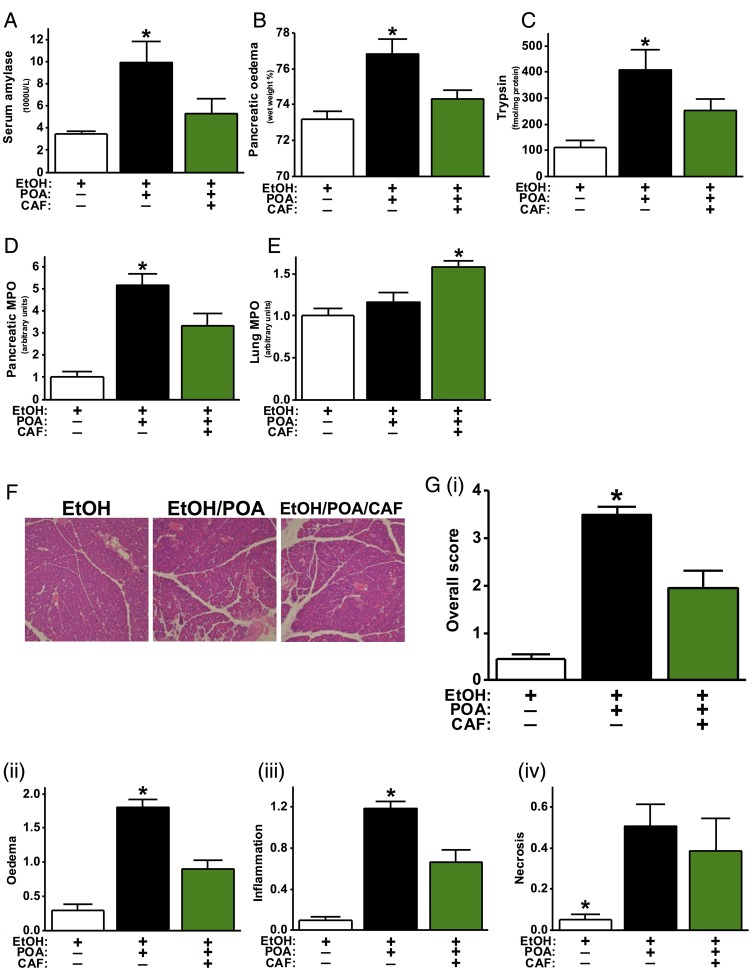
Protective effects of caffeine (CAF) on fatty acid ethyl ester acute pancreatitis. Mice received two intraperitoneal injections of ethanol (EtOH, 1.35 g/kg) in combination with palmitoleic acid (POA, 150 mg/kg) or equal amounts of EtOH injection only, 1 h apart. CAF at 25 mg/kg (seven injections hourly) was given 1 h after the second injection of EtOH/POA. Mice were sacrificed 24 h after disease induction and assessed for (A) serum amylase level, (B) pancreatic oedema, (C) pancreatic trypsin activity, (D) pancreatic myeloperoxidase (MPO) activity (normalised to EtOH group) and (E) lung MPO activity (normalised to EtOH group). (F) Representative pancreatic histopathology for all groups (H&E, ×200). (G) (i) Overall histopathological score and components: (ii) oedema, (iii) inflammation and (iv) necrosis. *p<0.05 vs other two groups. Values are means±SE of 10 animals per group.

Since caffeine inhibits FAEE-induced Ca^2+^ signals in vitro,^7^ its effects in FAEE-AP were tested. Co-administration of ethanol and POA caused typical AP features compared with ethanol alone (figure 8A–G).^7^ Two injections of 25 mg/kg caffeine significantly reduced serum amylase, pancreatic oedema, trypsin and MPO activity, although an increase in lung MPO activity was observed (figure 8A–E). The overall histopathological score (figure 8Gi) was greatly ameliorated, with significantly lowered oedema (figure 8Gii) and inflammation (figure 8Giii) with a trend towards a decrease in necrosis (figure 8Giv).

## Discussion

This study defines the inhibitory effects of methylxanthines on IP_3_R-mediated Ca^2+^ release from the pancreatic acinar endoplasmic reticulum store into the cytosol and their potential application in AP. It has been shown that caffeine inhibits IP_3_Rs^29^ as well as IP_3_ production in a concentration-dependent manner.^28^ We found that inhibition of IP_3_R-mediated Ca^2+^ release is attributable at least in part to an action on the IP_3_R, since xanthines inhibited IP_3_R-mediated Ca^2+^ release elicited by uncaged IP_3_. Caffeine, theophylline and paraxanthine prevented physiological Ca^2+^ signalling and toxic elevations of [Ca^2+^]_C_ induced by agents (CCK and TLCS) that cause AP, in a concentration-dependent manner (500 µM to 10 mM), also inhibiting falls in ΔΨ_M_ and necrotic cell death pathway activation. An inhibitory action on PDE preventing cAMP/cGMP degradation could not account for the effects on toxic [Ca^2+^]_C_ overload since additional cAMP/cGMP did not prevent these. Extending these findings in vivo, caffeine significantly reduced the severity of multiple, diverse models of AP. The combined concentrations of dimethylxanthine and trimethylxanthine after the 25 mg/kg caffeine protocol were within the range over which effects on both IP_3_R-mediated Ca^2+^ release and toxic elevations of [Ca^2+^]_C_ were identified. Despite the half-life of caffeine in mice of ∼60 min,^39^ the combined peak concentrations of dimethylxanthine and trimethylxanthine with the 25 mg/kg caffeine regimen were >2 mM, and serum caffeine was >400 μM 6 h after last caffeine injection. Following similar protocols of 25 mg/kg theophylline or paraxanthine, concentrations were far below the effective range on IP_3_Rs but within the effective range on PDE (approaching 100 µM 10 min after the last dimethylxanthine injection),^26^ and no protective effects on in vivo AP were seen. Nor were significant protective effects seen on pancreatic blood flow with the 25 mg/kg caffeine regimen, to be expected if mediated via PDE inhibition.^38^ Since pancreatic cellular injury initiates and determines severity in AP, the protective effect of caffeine on AP is likely to have been mediated by inhibition of IP_3_R-mediated Ca^2+^ release.

The concentration range over which caffeine inhibited toxic [Ca^2+^]_C_ overload induced by CCK hyperstimulation was similar to that seen here with quasi-physiological ACh-elicited Ca^2+^ oscillations, as previously in pancreatic acinar cells^28^ and permeabilised vascular smooth muscle cells.^40^ There could have been a cAMP/cGMP-dependent component to inhibition of the ACh-elicited Ca^2+^ oscillations since both xanthine-based and non-xanthine-based PDE inhibitors reduced ACh-elicited Ca^2+^ oscillations. Nevertheless, PDE inhibition is unlikely to have contributed to the reduction of toxic [Ca^2+^]_C_ overload as this was not affected by application of cell-permeable cAMP/cGMP analogues, but was immediately reversed upon caffeine administration. It is also unlikely that any increase in SERCA activity occurred in response to caffeine and downstream rises in cyclic nucleotide levels since no decrease in [Ca^2+^]_C_ was induced by analogues of cAMP and cGMP, which have been shown to upregulate SERCA via phospholamban.^41^ Therefore, the actions of caffeine on toxic [Ca^2+^]_C_ overload are consistent with a primary effect on IP_3_R-mediated Ca^2+^ release.

SOCE in pancreatic acinar and ductal cells occurs predominantly via Orai channels and is regulated in part by TRP channels.^42^ Previously we found inhibition of Orai to markedly reduce CER-AP, TLCS-AP and FAEE-AP.^15^ Inhibition of TRPC3 was found to reduce a mild model of CER-AP,^16^ while the non-selective cation channel TRPV1^43^ ^44^ as well as TRPA1^44^ have been implicated in neurogenic inflammation contributing to AP. We obtained no data to indicate any direct effect of caffeine on Orai or TRP channels. On the contrary, SOCE is unlikely to have been inhibited directly by caffeine since caffeine had no effect on thapsigargin-induced [Ca^2+^]_C_ plateaus, rather SOCE will have been inhibited secondarily to reduction of store depletion, the principal driver of SOCE in non-excitable cells.^14^ ^15^ ^21^

Inhibition of second messenger-mediated Ca^2+^ release via RyR ameliorates both caerulein^45^ and bile acid-induced AP.^46^ Since caffeine enhances Ca^2+^ release from RyRs in excitatory cells,^32^ and RyRs are major contributors to Ca^2+^ signalling in pancreatic acinar cells,^23^ ^47^ the effects of caffeine in the reduction of toxic Ca^2+^ overload observed here might appear contradictory. However, in contrast to the situation in muscle cells, caffeine can only release Ca^2+^ in pancreatic acinar cells under quite exceptional circumstances and then only when present at a low concentration (1 mM); indeed, this effect is abolished by stepping up the caffeine concentration.^29^ Furthermore, ACh-elicited Ca^2+^ signalling is blocked by inhibiting IP_3_Rs pharmacologically^29^ and knockout of the principal subtypes (IP_3_R2 and IP_3_R3) results in a failure of Ca^2+^ signal generation and secretion.^20^ Thus, caffeine is used extensively as an inhibitor of Ca^2+^ release in fundamental investigations of pancreatic acinar and other electrically non-excitable cells.^27^

Little, if any, protective effect of caffeine on experimental AP can be attributed to actions on adenosine receptors, which have both inhibitory (A1, A3) and excitatory (A2A, A2B) actions mediated in part through changes in cAMP.^48^ Caffeine is an antagonist of all adenosine receptors; the potency of caffeine is highest on A2A then A1 receptors at concentrations 10–20 times lower than on PDE.^26^ In the rat pancreas, few acinar cells express adenosine receptors;^49^ differential subtype expression occurs in vascular endothelium, nerve fibres, islet cells and ductal cells, with total expression A2A>A2B>A3>A1.^48^ While antagonism of the least predominant receptor (A1) previously reduced pancreatic oedema but no other parameter of experimental AP,^49^ the majority of data indicate that increasing adenosine receptor activation by reuptake inhibition or administration of A2 or A3 receptor agonists ameliorates experimental AP.^50^ Furthermore, adenosine receptor activation has broad anti-inflammatory effects, including reduction of neutrophil recruitment and effector functions via A2A and A2B;^51^ antagonism of these receptors may account for the lack of effect of caffeine on lung MPO or lung histopathology in experimental AP. Similarly, protective effects via adenosine receptors would be expected at doses of caffeine that had no (1 mg/kg) or minimal (5 mg/kg) effect.^52^

High doses of caffeine were required to reduce the severity of experimental AP, with the most effective 25 mg/kg regimen extending into toxicity, indicative of a very narrow therapeutic index. At this dose, the number of hourly injections had to be reduced from seven to two in FAEE-AP to avoid mortality; in CER-AP, 50 mg/kg resulted in caffeine intoxication syndrome, although at 25 mg/kg no visible side effects were observed. In humans, even 10 mg/kg caffeine would be likely to induce caffeine intoxication, with florid neuro-excitotoxic and other undesirable side effects.^26^ The principal caffeine metabolites in humans, monkeys, rabbits, rats and mice are similar and do not differ when given by mouth compared with intraperitoneally.^39^ Paraxanthine, however, is the most abundant dimethylxanthine metabolite in humans, while in mice this is theobromine.^39^ There is marked individual variability in caffeine metabolism and pharmacokinetics;^26^ since the half-life in humans typically ranges from 3 to 7 h, repeated high doses or continuous intravenous infusions would be hazardous unless rapid therapeutic monitoring were to be possible.

Our study has demonstrated proof of principle that caffeine causes marked amelioration of experimental AP, largely through inhibition of IP_3_R-mediated signalling. Medicinal chemistry starting with the template of caffeine and/or other compounds that inhibit IP_3_R-mediated signalling could lead to more potent, selective and safer drug candidates for AP.

## Supplementary Material

Web supplement
