# Supplementary material for: Caffeine protects against experimental acute pancreatitis by inhibition of inositol 1,4,5-trisphosphate receptor-mediated Ca2+ release
Source: Gut. 2015 Dec 7;66(2):301–13. doi: 10.1136/gutjnl-2015-309363 (PMC5284483; doi:10.1136/gutjnl-2015-309363)

SUPPLEMENTARY MATERIALS AND METHODS

Isolation of pancreatic acinar cells

Pancreatic acinar cells were freshly isolated from pancreata of young adult CD1 mice (8-12 week old) using a standard collagenase (Worthington Biochemical Corporation, Lakewood, NJ, USA) digestion procedure established in previous work.[6-10 S1] All experiments were performed at room temperature (23-25°C) unless otherwise indicated and cells were used within 4 h of isolation. The extracellular solution contained (mM): 140 NaCl, 4.7 KCl, 1.13 $MgCl_2$, 1 $CaCl_2$, 10 D-glucose, 10 HEPES. The final pH of the solution was adjusted to pH 7.35 using NaOH. For experiments longer than two hours, a modified solution was used. The standard physiological saline solution was supplemented with 1X minimal essential amino acid solution, 292 µg ml^{-1} L-glutamine, 100 µg ml^{-1} penicillin and streptomycin, 1 mg ml^{-1} soybean trypsin inhibitor, 1 mM $Na_3PO_4 \cdot 12H_2O$ and 1 mM pyruvate. The solution was sterilised by passing it once through a 200 nm filter (Appleton Woods, Selly Oak, UK).

Caffeine dose optimisation and other methylxanthine administration *in vivo*

To determine the optimal protocol of caffeine injections, we performed several preliminary experiments. We administered 100 mg/kg caffeine (7 intraperitoneal (i.p.) injections at hourly intervals), aiming to achieve a serum concentration of 5 mM, previously shown to inhibit toxin-induced elevations of the acinar cell cytosolic Ca^{2+} concentration ($[Ca^{2+}]_c$).[10, 23, 27 S2] Mice died at this dose, so the dose was reduced to 50 mg/kg, using the same injection protocol. At this dose mice survived but resulted in caffeine intoxication syndrome with irritability, increased urination and muscle twitching. The dose was reduced further to 25 mg/kg using the same protocol. At this

dose no neuro-excitotoxicity was observed. However, in the fatty acid ethyl ester-induced acute pancreatitis, 7 injections of 25 mg/kg caffeine (25 mg/kg regimen) resulted in several mouse fatalities so the injections were reduced to two injections, with the second of these one h after the first. 25 mg/kg theophylline or paraxanthine (7 i.p. injections at hourly intervals) resulted in no discernible side effects.

Serum sampling protocol for di- and trimethylxanthine assay in CER-AP

The 25 mg/kg caffeine regimen was given by injection at the same time as caerulein injections from the third caerulein injection in CER-AP, with two further caffeine injections at hourly intervals after the seventh caerulein injection. Serum samples were taken prior to the first caffeine injection, 10 min after the fourth caffeine injection and 10 min after the seventh caffeine injection as well as two and six hours after the seventh caffeine injection. Serum samples were then assayed by LC/MS for di- and trimethylxanthines. The same administration and sampling protocols were used to assay serum samples before and after theophylline or paraxanthine administration in CER-AP.

Serum amylase and serum IL-6 levels

Serum amylase was tested using a kinetic method by Roche automated clinical chemistry analyzers (GMI, Leeds, UK). Serum IL-6 levels were measured by the ELISA method using the protocols provided by R&D Systems (Abingdon, UK).

Pancreatic oedema

Pancreatic oedema was assessed by measuring pancreatic water content. A portion of pancreas (~60 mg) taken at the time of AP severity assessment was sliced, then

weighed and incubated at 90°C for 72 h. The fully dried tissue was weighed again and the pancreatic water content calculated as: wet weight-dry weight/wet weight ×100%.

Pancreatic trypsin activity

Pancreata were homogenised by a motorised homogeniser on ice in tissue buffer pH 6.5, containing (in mM) MOPS 5, sucrose 250 and magnesium sulfate 1. The homogenates were centrifuged at 1500 g for 5 min, and 100 µl of each supernatant was added to a cuvette containing the peptide substrate Boc-Gln-Ala-Arg-MCA (Peptide, Osaka, Japan) dissolved in 1900 µl pH 8.0 assay buffer containing (in mM) Tris 50, NaCl 150, $CaCl_2$ 1 and 0.1 mg/mL bovine serum albumin. Trypsin activity was measured by fluorimetric assay using a Shimadzu RF-5000 spectrophotometer (Milton Keynes, UK). Samples were excited at 380 nm and emissions collected at 440 nm[S2 S3]. A standard curve was generated using purified human trypsin. Pancreatic protein concentration was measured by a BCA protein assay (Thermo, Rockford, USA) using a BMG FlUOstar Omega Microplate Reader (Imgen Technologies, New York, USA). Trypsin activity was expressed as fmol/mg protein.

Myeloperoxidase activity

Myeloperoxidase activity in pancreas and lung was tested by a modified method from Dawra et al[S4]. Myeloperoxidase activity was measured by using the substrate 3,3′,5,5′-tetramethylbenzidine (TMB) in extracted supernatants from the samples. Briefly, 20 µl of the supernatant was added into the assay mix which consisted of 200 µL of phosphate buffer (100 mM, pH 5.4) with 0.5% HETAB and 20 µl TMB (20 mM in DMSO). This mixture was incubated at 37°C for 3 min, followed by addition of

50 µL H_2O_2 (0.01%). The mixture was further incubated for 3 min. The difference of absorbance between 0 min and 3 min at 655 nm was calculated against a standard curve generated by human myeloperoxidase using a plate reader.

Histopathology

Pancreatic and lung tissues were subjected to H&E staining and cut into 5 µm slides. For all experimental groups, 10 random fields of each pancreatic slide were graded by two independent blinded observers at magnification x200. Severity of pancreatic injury was defined by the extent of edema, inflammatory cell infiltration and acinar necrosis as previously described (each was scored as 0-3), and overall histopathology score calculated as the sum of individual scores.[10, 15] Lung histolopathology scores were determined by two independent blinded observers grading alveolar septal thickening in 10 random fields of each lung slide at magnification x200 (scored as 0-3 where 0 = normal, 1 = thickening <1/3 field, 2 = thickening ≥1/3 field and ≤ 2/3 field and 3 = thickening >2/3 field).

Measurement of pancreatic blood flow by fluorescent microsphere

To measure pancreatic blood flow, mice received either intraperitoneal injections of 50 µg/kg caerulein (CER, 7 injections hourly) or an equal number of equivalent volume saline injections. CAF (25 mg/kg) was given simultaneously with each CER injection. Mice were humanely killed 1 h after the last CER/CAF injection. The method to measure flow was adapted from that published by the Fluorescent Microsphere Resource Centre in the University of Washington (http://fmrc.pulmcc.washington.edu).[S5] In brief, male CD1 mice (35 g) had general anaesthesia induced and maintained with isofluorane. Under a dissecting

microscope, the left common carotid artery was accessed, ligated cranially and cannulated using 0.6 mm diameter polyethylene tubing. The tubing was advanced just beyond the thoracic inlet and secured using a bulldog clip. 200 IU of unfractionated sodium heparin was injected, followed by slow bolus injection of 0.5 ml phosphate buffered saline containing 140,000 red 10 μm diameter polystyrene microbeads (FluoSpheres, Red (580/605), Molecular Probes Europe, Leiden, The Netherlands) over 60 seconds. Animals were culled by intravascular injection of pentobarbital and organs (pancreas, lung, kidneys) harvested immediately. Whole organs were placed in 1.5 ml Eppendorf tubes and weighed prior to further processing.

36,000 blue-green polystyrene microspheres (430/465) were added to each Eppendorf tube as procedural controls and organs digested in 1 ml of ethanolic KOH for 48h at 50 °C, vortexing at 24 and 48 h. Tubes were then centrifuged at 2000 g for 20 min and pellets resuspended in distilled water with 1% Triton-100, vortexed and again centrifuged at 2000 g for 20min. Pellets were resuspended in phosphate buffer (pH 7), vortexed and centrifuged before resuspending in 1ml of 2-ethoxy-ethyl acetate, vortexed and kept in a dark at room temperature for 24 h. Samples were then centrifuged at 2000 g for 20min and fluorescence of supernatants determined using the BMG FlUOstar Omega Microplate Reader (BMG LABTECH, Aylesbury, UK), sequentially measuring red (420/460) and blue-green fluorescence (580/620).

Individual experimental results were excluded if blue-green fluorescence differed by >20% from average, indicating loss of spheres during processing. Results were also excluded if the difference in red fluorescence between left and right kidneys was

>20%, indicating inadequate mixing of spheres prior to injection. Fluorescent values per weight of pancreas were compared between experimental groups, with n ≥ 4 experimental repeats per group.

SUPPLEMENTARY FIGURE LEGENDS

Supplementary Figure 1. Effect of monomethylxanthine and xanthine on ACh-induced Ca^{2+} oscillations in isolated pancreatic acinar cells. **(A)** 1-methylxanthine (1-MX, 2 mM) had minimal inhibitory effect on ACh-induced Ca^{2+} oscillations, while **(B)** Xanthine (2 mM) did not show any effect. Traces are averages from >20 cells from at least three repeat experiments. Data are normalised from basal fluorescence levels (F/F_0).

Supplementary Figure 2. Effect of caffeine (CAF) on CCK- and TLCS-induced Ca^{2+} plateaus in isolated pancreatic acinar cells. **(A)** CAF (10 mM) nearly abolished CCK-induced Ca^{2+} plateaus. (B) CAF (10 mM) converted TLCS-induced Ca^{2+} plateaus into oscillations. Traces are averages from >20 cells from at least three repeat experiments. Data are normalised from basal fluorescence levels (F/F_0).

Supplementary Figure 3. Phosphodiesterase inhibitors block ACh-induced Ca^{2+} oscillations in isolated pancreatic acinar cells. **(A)** Non-selective phosphodiesterase inhibitors methylxanthines (MXs) - caffeine (CAF), theophylline (TP), paraxanthine (PX), theobromine (TB), 1-MX, 7-MX and 3-isobutyl-1-methylxanthine (IBMX) showed significant dose-dependent inhibition at 500 µM and 2 mM. TP (2 mM) showed the greatest inhibition that was similar to the IBMX positive control. Mono-MXs showed the least inhibition, and no significant inhibition was detected with 500

µM 7-MX (*p <0.05 vs control; †p <0.05 vs lower concentration). **(B)** IBMX (2 mM) and **(C)** a synthesised MX derivative pentoxfylline (PTX, 2 mM) were shown to have a complete inhibitory effect on ACh-induced Ca^{2+} oscillations. **(D)** (i) No significant inhibitory effect was observed when rolipram (ROL), a selective non-xanthine based phosphodiesterase 4 inhibitor, at 100 µM was used. (ii) A marked inhibition, however, was achieved when the concentration of ROL was increased to 200 µM. Traces are averages of >20 cells from at least three repeat experiments. Data are normalised from basal fluorescence levels (F/F_0) and expressed as means ± SE in histograms.

Supplementary Figure 4. Effects of caffeine (CAF) on lung histopathology, renal function, renal histology and pancreatic blood flow in CER-AP. **(A)** Representative images of lung histopathology for in controls, CER-AP and CER-AP with caffeine (H&E, ×200) and **(C)** lung histopathology scores. **(D)** Serum creatinine and **(E)** representative images of kidney histology for all groups and caffeine alone (H&E, ×200). (E) Pancreatic blood flow in all three groups. (*p <0.05 vs other groups. Values are means ± SE of 4-6 animals per group).

Supplementary Figure 5. Dose response of dimethylxanthines on the severity of CER-AP at 12 h. Mice received intraperitoneal injections of 50 µg/kg caerulein (7 injections hourly) and either theophylline (TP) or paraxanthine (PX) at either 10 and 25 mg/kg regimens (7 injections hourly) was given 2 h after the first injection of caerulein. Control groups received the same volume of saline injections from 2 h after the first injection of caerulein. Mice were humanely killed at 12 h after disease induction and assessed by **(A)** Serum amylase, **(B)** Pancreatic oedema, **(C)**

Pancreatic trypsin activity and **(D)** Pancreatic myeloperoxidase (MPO) activity (normalised to saline group). **(E)** (i) Overall histopathological score and breakdown of components: (ii) oedema, (iii) inflammation and (iv) necrosis. *$p<0.05$ vs saline group. Values are means ± SE of 6 animals per group.

Supplementary Figure 6. Determination of dimethylxanthine levels in CER-AP. Mice received either intraperitoneal injections of 50 µg/kg caerulein (7 injections hourly). Theophylline (TP) or paraxanthine (PX) at either 10 or 25 mg/kg regimen (7 injections hourly) was given respectively 2 h after the first injection of caerulein. Mice were humanely killed at 12 h after disease induction and serum dimethylxanthine levels assessed by LC/MS. **(A)** Serum mean levels of TP were 18.1 and 67.4 µM for 10 and 25 mg/kg TP regimens, respectively. **(B)** Mean serum levels of PX were 48.0 and 91.0 µM for 10 and 25 mg/kg PX regimens, respectively. Values are means ± SE of 6 animals per group.

SUPPLEMENTARY REFERENCES

S1. Booth DM, Murphy JA, Mukherjee R, *et al.* Reactive oxygen species induced by bile acid induce apoptosis and protect against necrosis in pancreatic acinar cells. *Gastroenterology* 2011;140:2116-25.

S2. Nathan JD, Romac J, Peng RY, *et al.* Transgenic expression of pancreatic secretory trypsin inhibitor-I ameliorates secretagogue-induced pancreatitis in mice. *Gastroenterology* 2005;128:717-27.

S3. Kawabata S, Miura T, Morita T, *et al.* Highly sensitive peptide-4-methylcoumaryl-7-amide substrates for blood-clotting proteases and trypsin. *FEBS J* 1988;172:17-25.

S4. Dawra R, Ku YS, Sharif R, *et al.* An improved method for extracting myeloperoxidase and determining its activity in the pancreas and lungs during pancreatitis. *Pancreas* 2008;37:62-8.

S5. Manual for using fluorescent microspheres to measure regional organ perfusion. Seattle, WA: University of Washington, 1999.

Figure S1

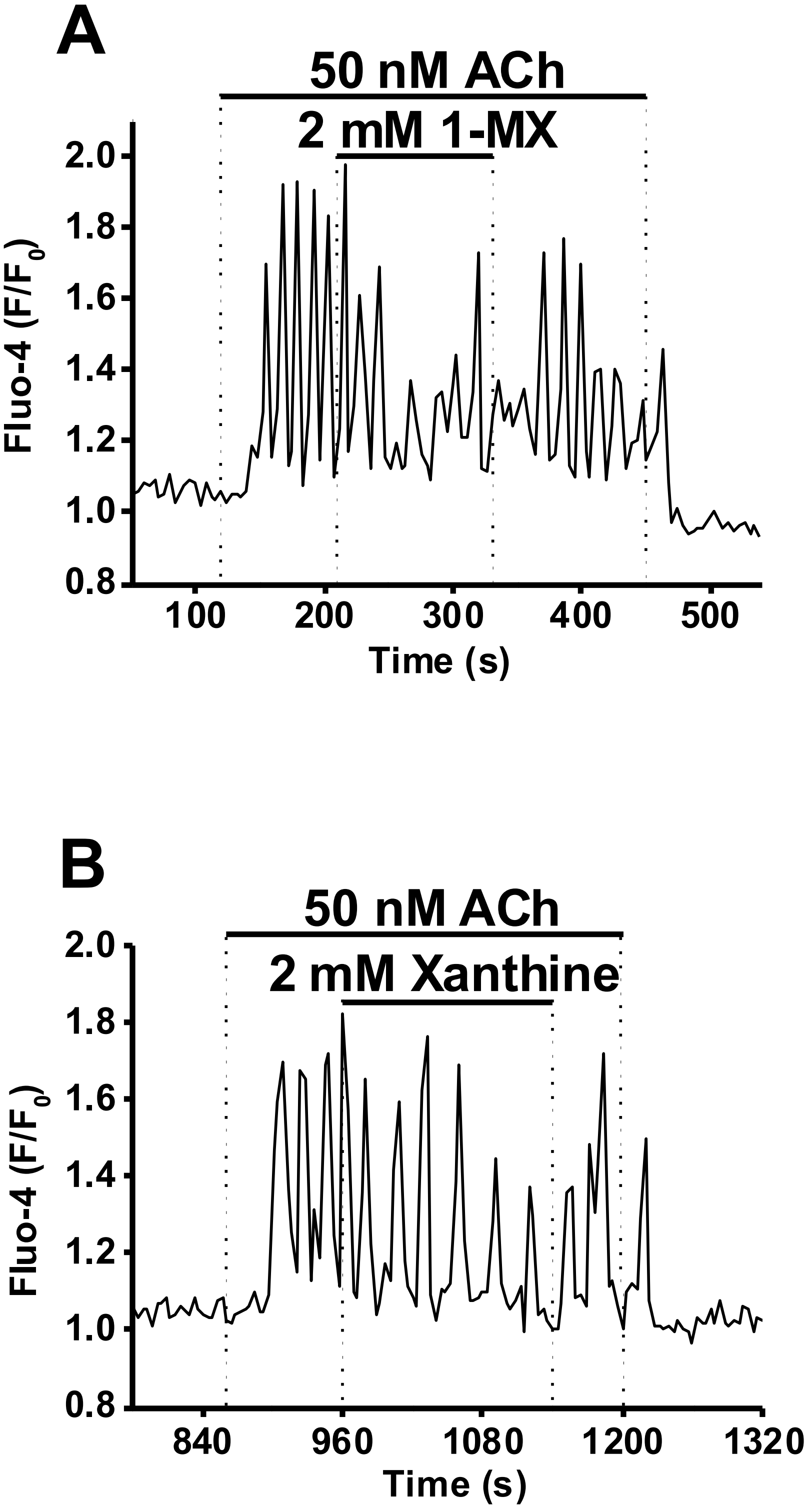
A
50 nM ACh
2 mM 1-MX
Fluo-4 (F/F$_0$)
2.0
1.8
1.6
1.4
1.2
1.0
0.8
100
200
300
400
500
Time (s)
B
50 nM ACh
2 mM Xanthine
Fluo-4 (F/F$_0$)
2.0
1.8
1.6
1.4
1.2
1.0
0.8
840
960
1080
1200
1320
Time (s)

Figure S2

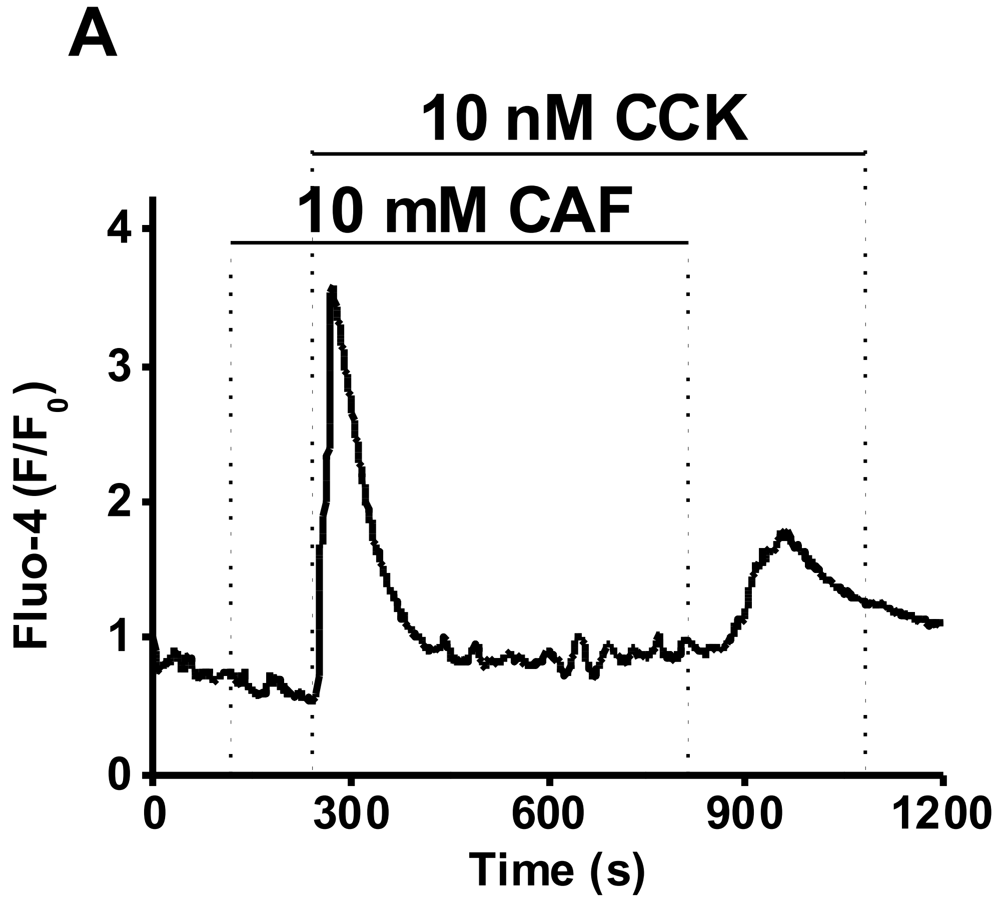

A
10 nM CCK
10 mM CAF
Fluo-4 (F/F0)
0
1
2
3
4
0
300
600
900
1200
Time (s)

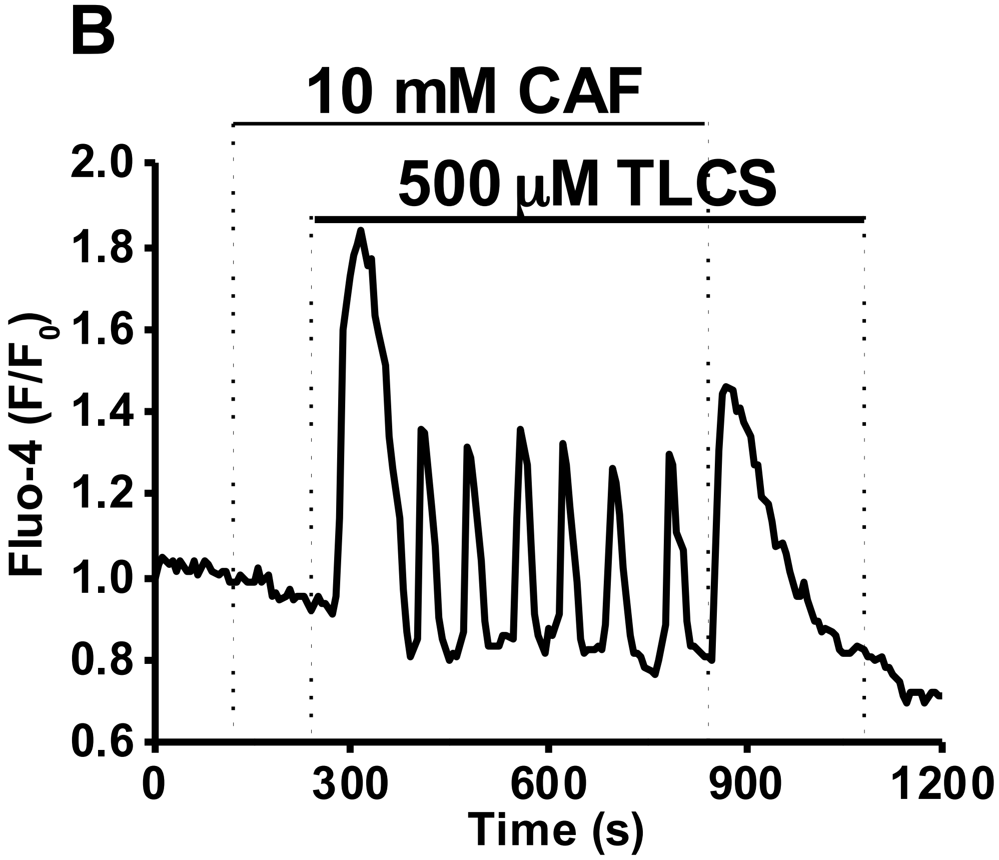

B
10 mM CAF
500 μM TLCS
Fluo-4 (F/F0)
0.6
0.8
1.0
1.2
1.4
1.6
1.8
2.0
0
300
600
900
1200
Time (s)

Figure S3

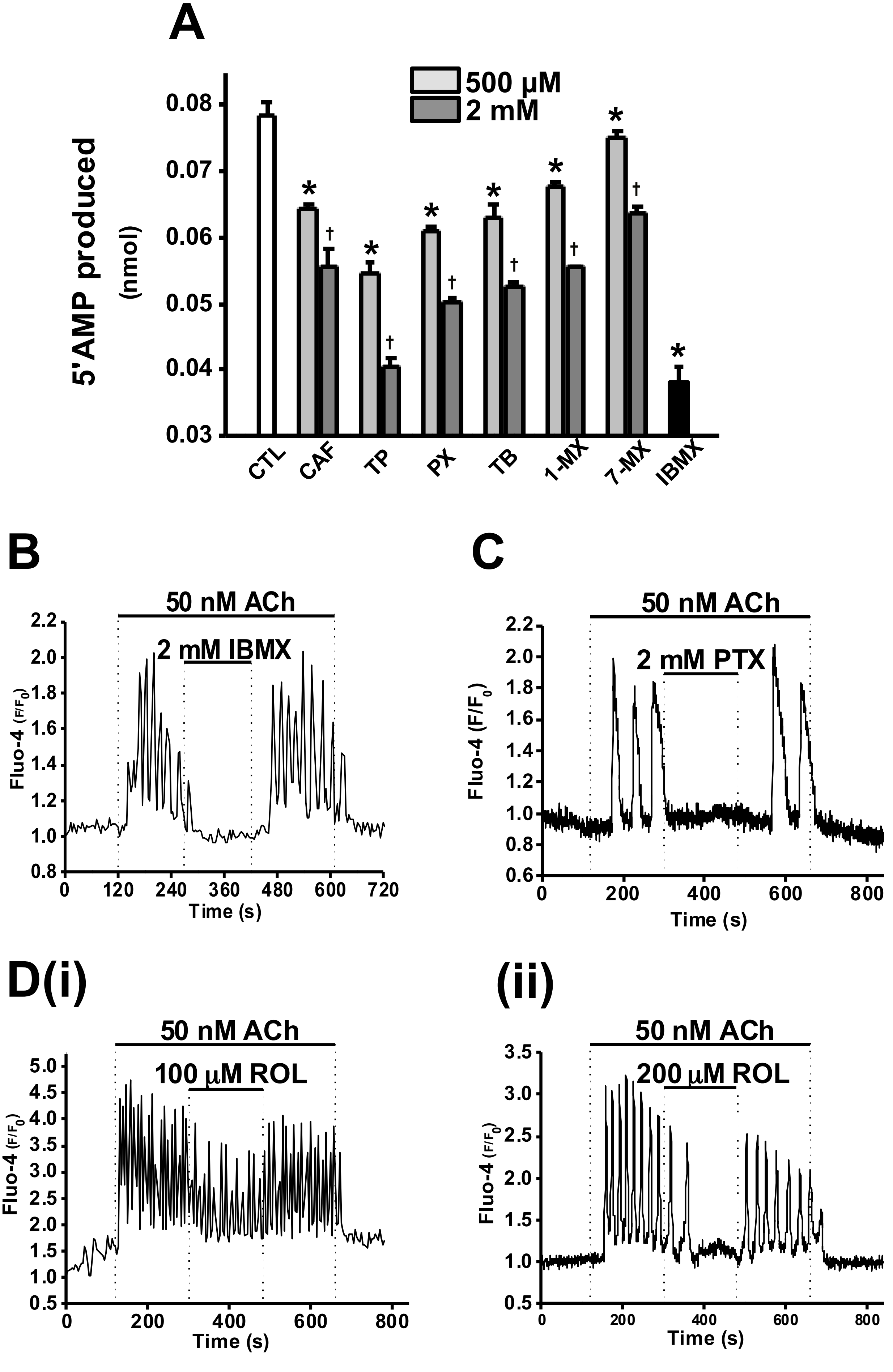

A
500 µM
2 mM
5'AMP produced
(nmol)
0.08
0.07
0.06
0.05
0.04
0.03
CTL
CAF
TP
PX
TB
1-MX
7-MX
IBMX
B
50 nM ACh
2 mM IBMX
Fluo-4 (F/F0)
Time (s)
C
50 nM ACh
2 mM PTX
Fluo-4 (F/F0)
Time (s)
D(i)
50 nM ACh
100 µM ROL
Fluo-4 (F/F0)
Time (s)
(ii)
50 nM ACh
200 µM ROL
Fluo-4 (F/F0)
Time (s)

Figure S4

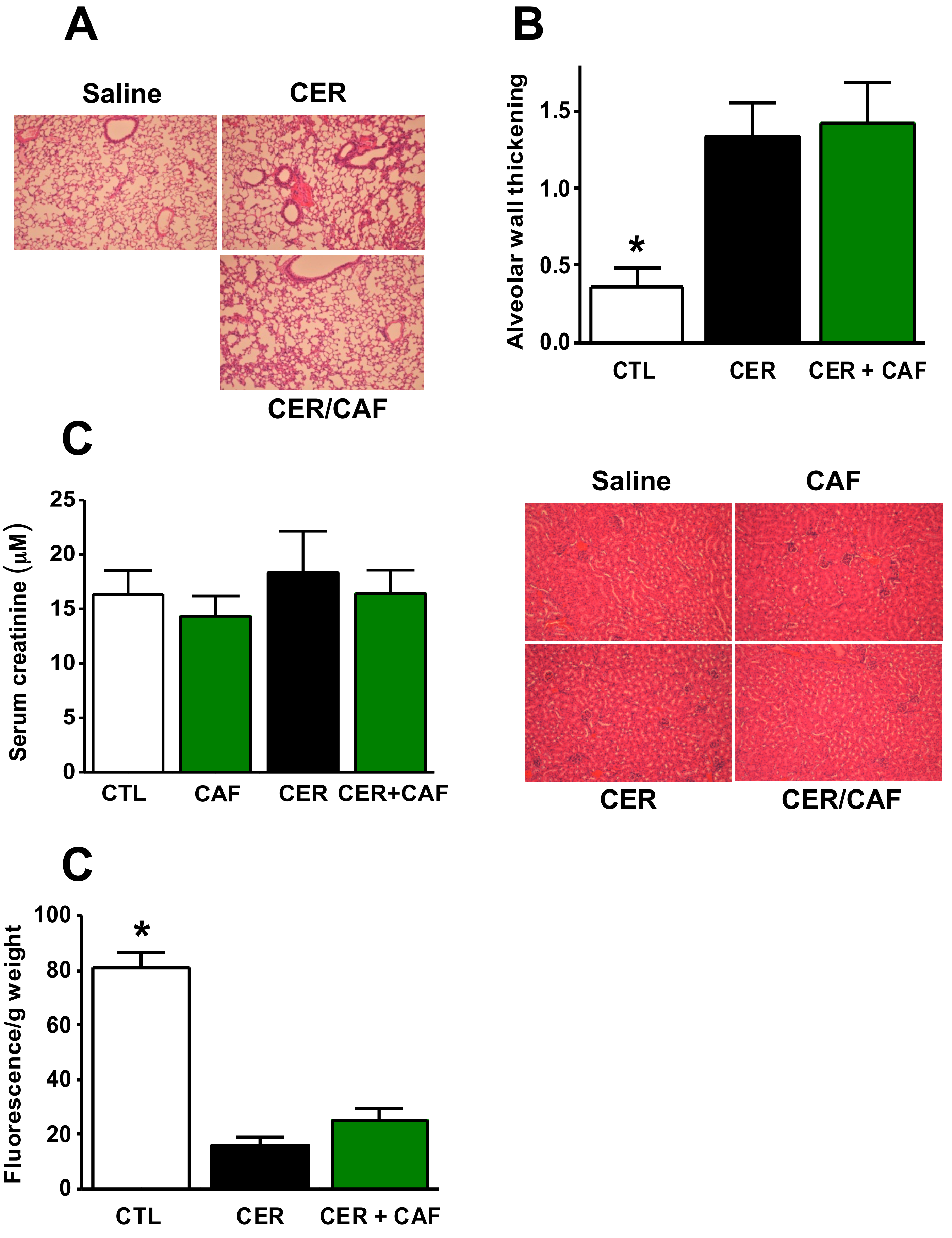
A
Saline
CER
CER/CAF
B
Alveolar wall thickening
0.0
0.5
1.0
1.5
*
CTL
CER
CER + CAF
C
Serum creatinine (μM)
0
5
10
15
20
25
CTL
CAF
CER
CER+CAF
Saline
CAF
CER
CER/CAF
C
Fluorescence/g weight
0
20
40
60
80
100
*
CTL
CER
CER + CAF

Figure S5

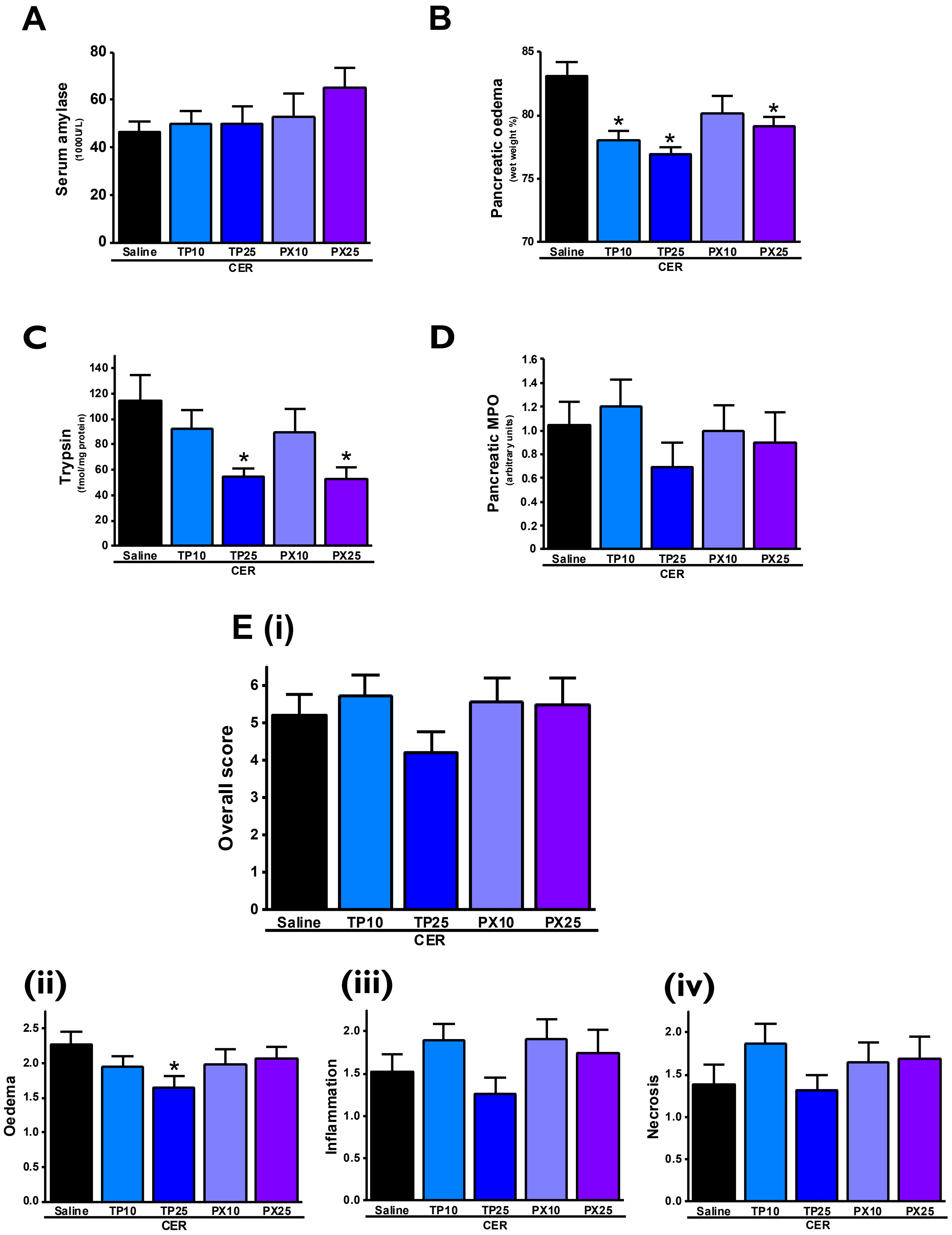
A
Serum amylase
(1000U/L)
0
20
40
60
80
Saline
TP10
TP25
PX10
PX25
CER
B
Pancreatic oedema
(wet weight %)
70
75
80
85
*
*
*
Saline
TP10
TP25
PX10
PX25
CER
C
Trypsin
(fmol/mg protein)
0
20
40
60
80
100
120
140
*
*
Saline
TP10
TP25
PX10
PX25
CER
D
Pancreatic MPO
(arbitrary units)
0
0.2
0.4
0.6
0.8
1.0
1.2
1.4
1.6
Saline
TP10
TP25
PX10
PX25
CER
E (i)
Overall score
0
1
2
3
4
5
6
Saline
TP10
TP25
PX10
PX25
CER
(ii)
Oedema
0.0
0.5
1.0
1.5
2.0
2.5
*
Saline
TP10
TP25
PX10
PX25
CER
(iii)
Inflammation
0.0
0.5
1.0
1.5
2.0
Saline
TP10
TP25
PX10
PX25
CER
(iv)
Necrosis
0.0
0.5
1.0
1.5
2.0
Saline
TP10
TP25
PX10
PX25
CER

Figure S6

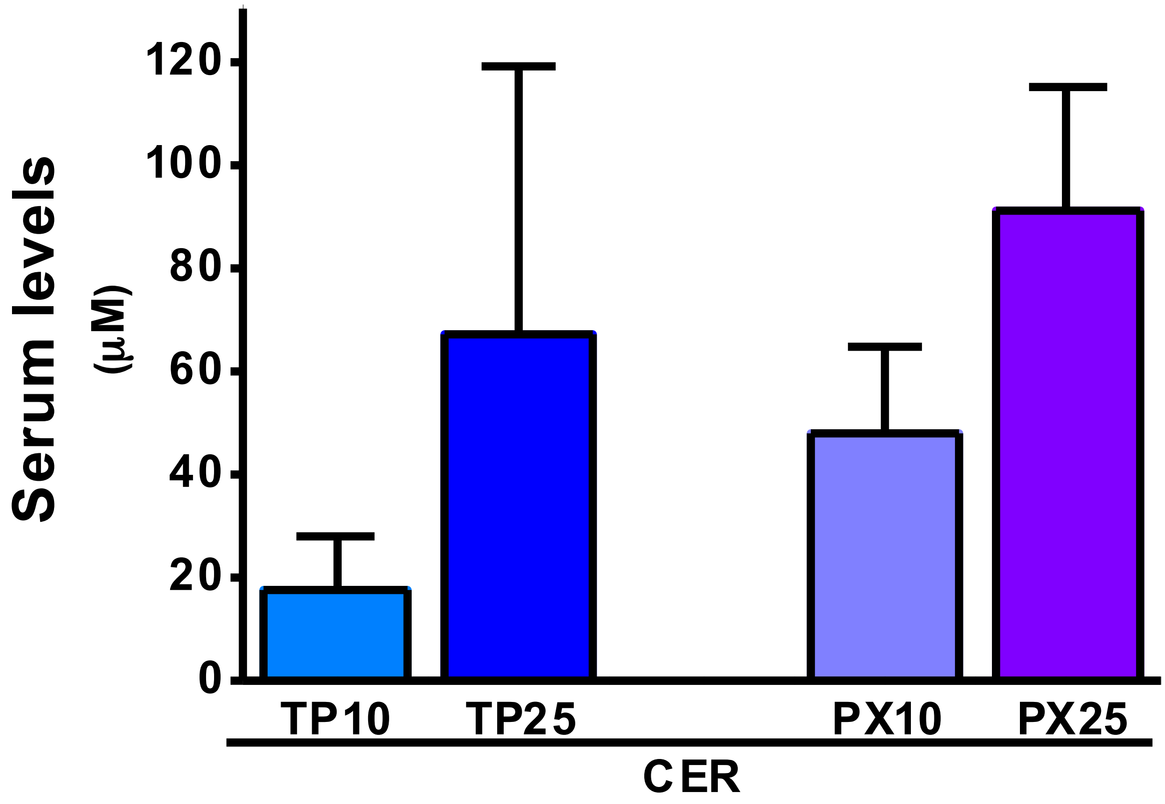

Supplement: Web supplement [file gutjnl-2015-309363-s1.pdf]
